# ﻿Preliminary study of marine sponges (Porifera) in the littoral of Spermonde Archipelago, Indonesia

**DOI:** 10.3897/zookeys.1208.113603

**Published:** 2024-08-01

**Authors:** Singgih Afifa Putra, Rohani Ambo-Rappe, Jamaluddin Jompa, Nicole J. de Voogd

**Affiliations:** 1 Universitas Hasanuddin, Fakultas Ilmu Kelautan dan Perikanan, Program Doktor Ilmu Perikanan, Makassar 90245, Sulawesi Selatan, Indonesia; 2 Balai Pengembangan Penjaminan Mutu Pendidikan Vokasi Bidang Kelautan Perikanan Teknologi Informasi dan Komunikasi (BPPMPV KPTK), Gowa 92172, Sulawesi Selatan, Indonesia; 3 Universitas Hasanuddin, Fakultas Ilmu Kelautan dan Perikanan, Program Studi Ilmu Kelautan, Makassar 90245, Sulawesi Selatan, Indonesia; 4 Naturalis Biodiversity Center, Understanding Evolution Group, 2333 CR Leiden, Netherlands; 5 Leiden University, Institute of Biology Leiden (IBL), Sylviusweg 72, 2333 BE Leiden, Netherlands

**Keywords:** Calcarea, Demospongiae, Indo-Pacific, taxonomy, turbid habitats

## Abstract

Previous ecological studies show higher sponge diversity in the Spermonde Archipelago, SW Sulawesi, Indonesia, compared to the World Porifera Database. This study aims to provide an updated checklist of sponges of the Spermonde Archipelago, focusing particularly on the littoral area. Systematic sampling was executed through several observations, with roving techniques, e.g., snorkeling and SCUBA diving. In situ photographs of living sponges were taken using an underwater digital camera. Some specimens were collected and stored at the Naturalis Biodiversity Center, Leiden. Fragments of samples were analyzed using light and scanning electron microscopy. A total of 27 sponges (Calcarea and Demospongiae) were catalogued from the littoral area of the Spermonde Archipelago. Some of these are new records for the Sulawesi Sea/Makassar Strait marine ecoregion, including four potentially novel taxa. Preliminary morphological descriptions of all examined samples are presented. This study highlights the sponge assemblage flourishing in a shallow area characterized by a paucity of live corals and a predominant environment by macroalgae, rocks, and rubble.

## ﻿Introduction

The Spermonde Archipelago is located between the south-western part of Sulawesi and the Makassar Strait in Indonesia (Kench and Mann 2017). This region is placed in the Sulawesi Sea/Makassar Strait (SS/MS) marine ecoregion based on Marine Ecoregions of the World (Spalding et al. 2007). The whole archipelago consists of many coral cays and small islands (Umbgrove 1928; Kench and Mann 2017), with the highest coral cover less than 60% (Sari et al. 2021). The coral reef is the richest ecosystem with high species diversity (Cairns 1999; Williams et al. 2019; Kusumoto et al. 2020). Every part of the reef is influenced by different regimes of wave actions, light intensity, bathymetric range, and water currents (Kench and Mann 2017). The sponge community is one of the essential components of the reef environment (Rützler 2004), showing a wide distribution across the Spermonde Archipelago (de Voogd et al. 1999). This community is also recognized as comprising predominantly niche specialists with marked habitat preferences in coral reef ecosystems (Hooper 2008).

Numerous studies have been conducted on this archipelago due to its geological, biodiversity, and ecological significance in marine biology (Polónia et al. 2015). Taxonomic studies on sponge diversity in this region were sporadic. The sponge fauna within the SS/MS marine ecoregion is relatively well studied only in north Sulawesi (de Voogd et al. 2024). Only a few papers have conducted morphological taxonomic studies to describe new species or revise specific group of sponges (e.g., genus, family, or order), with a mention of the Spermonde Archipelago as a locality (de Weerdt and van Soest 2001; de Voogd 2004; Becking 2013; Alvarez et al. 2016; van Soest et al. 2021).

Globally, more than 9,000 sponge species are currently described (de Voogd et al. 2024). Taxonomic misidentifications by non-taxonomists are common when dealing with sponges (Cárdenas et al. 2022). Some comprehensive inventories of the sponge fauna from Indonesia have been published (van Soest 1990; Calcinai et al. 2017), including specific sponge category-based inventories (de Voogd and van Soest 2002; Calcinai et al. 2005; van Soest and de Voogd 2015; van Soest et al. 2021). However, sponge diversity across the Indonesian Archipelago is still considered underestimated (Calcinai et al. 2017; Putra et al. 2023).

According to the World Porifera Database (de Voogd et al. 2024), sponge diversity in the Sulawesi Sea/Makassar Strait marine ecoregion comprises 128 species, i.e., 17 species of Calcarea, 97 species of Demospongiae,13 species of Hexactinellida, and one species of Homoscleromorpha. The class Demospongiae is predominantly represented by the order Poecilosclerida, comprising 31 species. However, the latest ecological study reveals a higher sponge species beta diversity in the Spermonde Archipelago, SW Sulawesi. At least 151 species belonging to 68 genera and 37 families were identified in this area (de Voogd et al. 2006). Therefore, taxonomic studies are needed to describe the unregistered sponge species and elucidate the sponge alpha diversity in this marine ecoregion.

The current study is focused on the littoral area of the archipelago. This area is below the lowest tide, but including the reef flat. Reef flats are the most recent expression of sea-level coral reef growth (Hopley 2011). This area presents extreme conditions for coral reefs due to marginal environmental factors (Burt et al. 2020). Furthermore, the coral reef ecosystem in this shallow area, particularly in the inner zone of the archipelago, was reported to be in a very poor condition, ≈ 5–14% (Parenden et al. 2021; Sari et al. 2021). This habitat is dominated by dead corals with algae, macroalgae, and sediment cover (Parenden et al. 2021).

This study aims to provide preliminary morphological identifications of sponge specimens from the Spermonde Archipelago to fill the knowledge gap concerning marine sponge diversity of Indonesia. Additionally, it seeks to promote the study of sponge taxonomy in Indonesia and to update the checklist of sponge diversity of this marine ecoregion.

## ﻿Materials and methods

### ﻿Specimen collection

**Figure 1.**
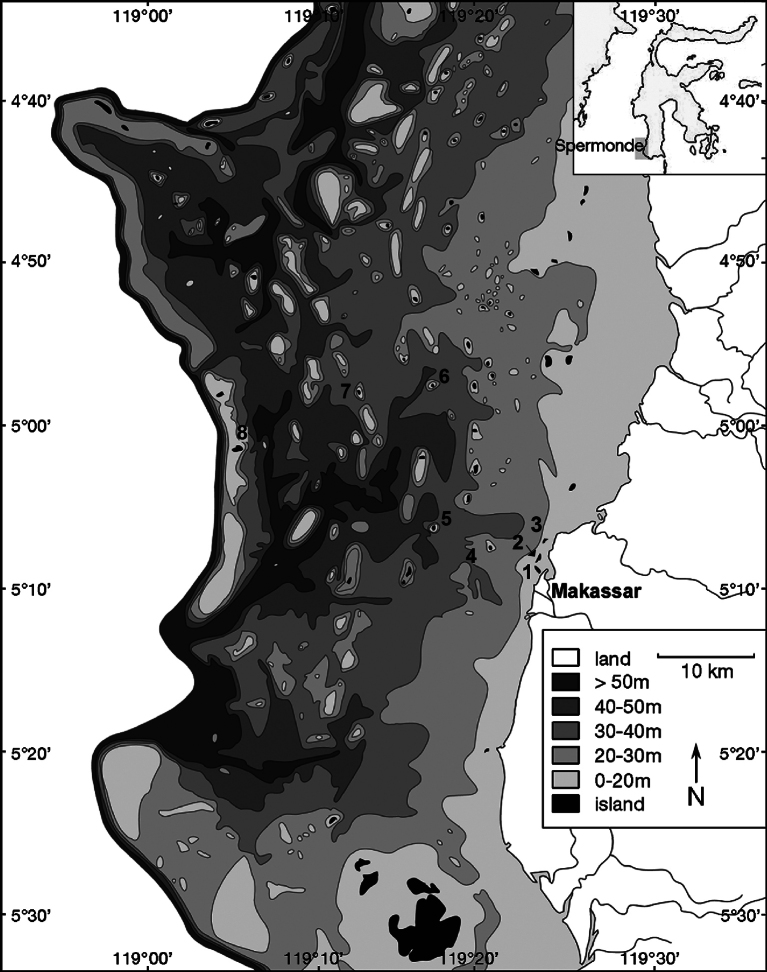
The location of sponge observation in the shallow-subtidal area of the Spermonde Archipelago, SW Sulawesi, Indonesia, i.e., 1) Lae-lae, 2) Gusung (as Gusung Tallang), 3) Kayangan, 4) Samalona, 5) Kudingarengkeke, 6) Badi, 7) Lumulumu, 8) Langkai.

The specimen collection was conducted through several observations of the littoral area of the Spermonde Archipelago, Indonesia. Some observations were made by NJdeV in 2018, and by SAP during 2020 and 2021 (Fig. 1, Suppl. material 1). The observations were performed using a roving technique (Pattengill-Semmens 2001) through snorkeling or SCUBA Diving. Roving time is 1–2 hours within ≈ 90 m^2^ for each site. The timed survey method does not provide density and abundance data but is most useful when the study aims to assess biodiversity (Reimer et al. 2018; Montano et al. 2020).

Photographs of living sponges at the study site (in situ) were captured using an underwater digital camera (Nikon Coolpix W300 and Olympus TG-series). The specimens were immediately transferred into 96% ethyl alcohol for preservation during observation (Hooper 2003), and some of them were deposited in the museum collection of the Naturalis Biodiversity Center, Leiden, The Netherlands (**NBC**); the others are located at Balai Pengembangan Penjaminan Mutu Pendidikan Vokasi Bidang Kelautan Perikanan Teknologi Informasi dan Komunikasi (**BPPMPV KPTK**) in Gowa, Sulawesi Selatan.

### ﻿Specimen identification

Fragments of sponges and sections of the skeleton were prepared and then examined using light microscopy (Leica DM5500 B and Olympus BX53) and JEOL Scanning Electron Microscope (JSM-6480LV) at the Naturalis Biodiversity Center, Leiden, following standard procedures for skeleton and spicule analysis (Rützler 1974; Boury-Esnault and Rützler 1997; Hooper 2003). Except for macro morphologies, which were measured with a vernier caliper, microscopic characteristics were assessed using Olympus cellSens Standard and Leica LAS Core software. Images were cleaned up and assembled in composite figures using Adobe Photoshop 2023 and Adobe Illustrator 2023 licensed to SAP. Measurements of spicules (smallest-largest-(mean)) rely on a minimum of 20 measurements of length and thickness for each type of spicule in the case of one or a few specimens. Systematic treatment refers to the description of Porifera morphological identification (Hooper and van Soest 2002) and the World Porifera Database/WPD (de Voogd et al. 2024). The recording of species names includes as much information as possible, such as valid names, species location, specimen description, and other taxonomic notes.

## ﻿Results

### ﻿Systematics

Accepted names, all synonyms, and systematic updates were based on the World Porifera Database (de Voogd et al. 2024), and all terminology follows updated terms (Boury-Esnault and Rützler 1997; Łukowiak et al. 2022).

#### ﻿Phylum Porifera Grant, 1835

**Class Calcarea Bowerbank, 1862**

**Subclass Calcinea Bidder, 1898**

**Order Clathrinida Hartman, 1958**

**Family Clathrinidae Minchin, 1900**

**Genus *Clathrina* Gray, 1867**

##### ﻿ Clathrina rodriguesensis

Taxon classificationAnimaliaClathrinidaClathrinidae

van Soest & de Voogd, 2018

5089ECE8-E915-5F73-973F-8871A7CC85F6

Fig. 2

###### Diagnostic features.

In its natural environment, the species forms a large, encrusting mass composed of wide, closely linked tubes showing little variation in diameter. According to van Soest and de Voogd (2018), it can spread flatly across wide areas, with the tubes arranged like a ladder. The main tubes often end in an opening slightly raised from the mass. The color is white with shades of blue, grey, or pink, turning pale beige or brown when preserved. Consistency firm and with asconoid aquiferous system. Spicules are only triactines.

**Figure 2.**
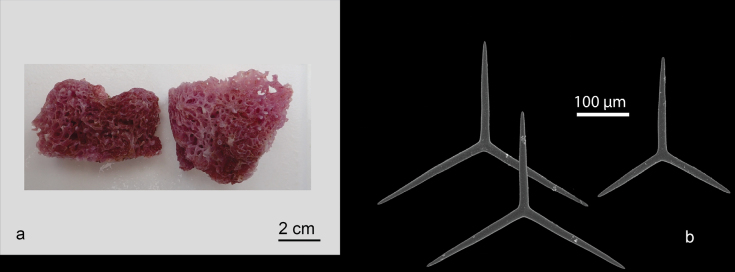
*Clathrinarodriguesensis* van Soest & de Voogd, 2018 from Kudingareng keke Island, the Spermonde Archipelago (Sample CEL035) **a** habitus of fresh specimen (photograph by NJdeV) **b** SEM image of the triactines.

###### Distribution and ecology.

Previously, this species only recorded from Seychelles, Western Indian Ocean (van Soest and de Voogd 2018). This is first record for Indonesia (Kudingareng keke, the Spermonde Archipelago; reef flat).

#### ﻿Genus *Janusya* Klautau et al., 2021

##### ﻿ Janusya tubuloreticulosa

Taxon classificationAnimaliaClathrinidaClathrinidae

(van Soest & de Voogd, 2015)

580ECFDD-832C-5754-9E9A-A6ABDBBE9262

Fig. 3

###### Diagnostic features.

An orange flattened mass of short oscular tubes, connected at the substratum by a basal tubular network, the erect tubes maybe divided into one or two side tubes. The walls of tubes are thin with spicules are dominated by triactines. Triactines predominantly equiactinal with size 14.93–120.79 (83.54) × 3.39–6.76 (5.48) µm (*n* = 20). Tetractines are also not rarely found with size 28.04–103.79 (83.77) × 4.98–5.94 (5.48) µm (*n* = 11).

**Figure 3.**
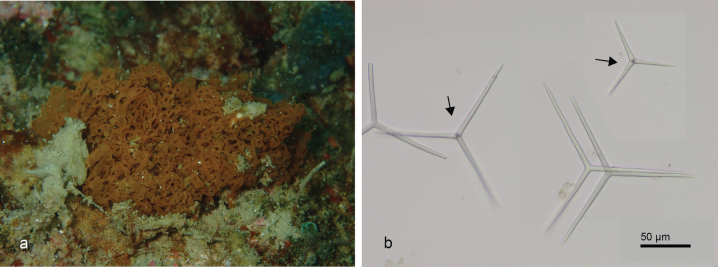
*Janusyatubuloreticulosa* (van Soest & de Voogd, 2015) from Samalona Island, the Spermonde Archipelago (Sample CEL001) **a** habitus in situ at Samalona reefs (photograph by NJdeV) **b** LM images of spicules, triactines and tetractines (arrows).

###### Distribution and ecology.

Originally reported from Ternate (van Soest and de Voogd 2015). First record from Samalona Island, the Spermonde Archipelago; reef flat.

#### ﻿Family Leucaltidae Dendy & Row, 1913

**Genus *Laucaltis* Haeckel, 1872**

##### ﻿ Leucaltis nodusgordii

Taxon classificationAnimaliaClathrinidaLeucaltidae

(Poléjaeff, 1883)

F2B0CE38-5BE0-51FD-B6BC-5E509A64C28E

Fig. 4

###### Diagnostic features.

The species forms a clathrate mass of interconnected (anastomosing) tubes with varying lengths and diameters. Individual tubes can reach up to 2.5 cm in length and have diameters of 2–8 mm (van Soest and de Voogd 2015). The tubes end in oscula, which can be as wide as the tube itself (standing upright) or smaller (flush with the surface), and these oscula are naked. The surface is smooth, and the texture is brittle yet somewhat compressible. The color is white or pinkish white, sometimes lavender-colored, and it turns yellowish white when preserved. The cortical skeleton is formed by the basal triradiate system of giant tetractines mixed with giant triactines. Actines of the giant tetractines and triactines protrude into the choanosomal skeleton. Next to the actines of the giant tri- and tetractines, the choanosomal skeleton contains scattered intermediate to small-sized regular triactines and tetractines (see van Soest and de Voogd 2015 for detail description).

**Figure 4.**
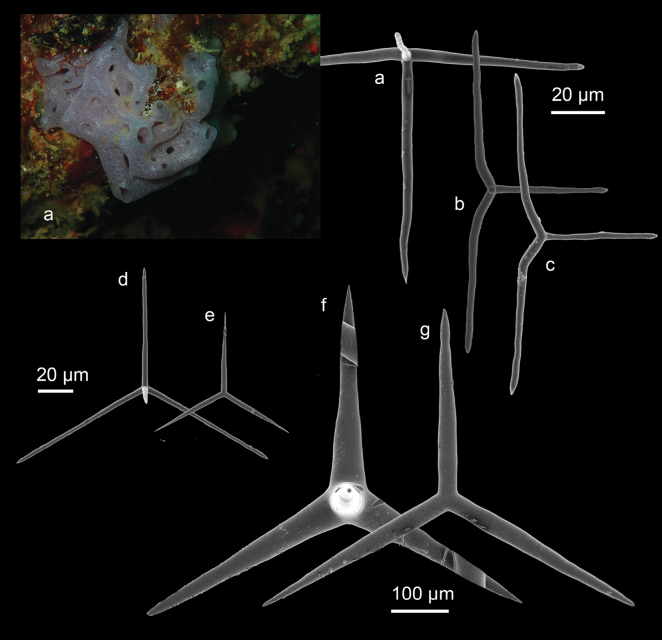
**a** habitus in situ *Leucaltisnodusgordii* (Poléjaeff, 1883) (CEL005) from Samalona Island, the Spermonde Archipelago (photograph by NJdeV). SEM images of spicules **a** regular equiangular tetractine of the chamber laye **b** ‘Abruptly angled’ tetractines **c** ‘Abruptly angled’ triactines (**b, c** both from the atrial region) **d** Small regular-shaped tetractines of the chamber layer **e** small regular-shaped triactines of the cortical region **f** Giant sized tetractines **g** giant sized triactines (**f, g** both from the cortical region).

###### Distribution and ecology.

*Leucaltisnodusgordii* is a new record for the Spermonde Archipelago (Samalona Island); reef flat. This species has been reported previously from north Sulawesi (van Soest and de Voogd 2015).

#### ﻿Class Demospongiae Sollas, 1885

**Subclass Heteroscleromorpha Cárdenas et al., 2012**

**Order Clionaida Morrow & Cárdenas, 2015**

**Family Spirastrellidae Ridley & Dendy, 1886**

**Genus *Spirastrella* Schmidt, 1868**

##### ﻿ Spirastrella aff. decumbens

Taxon classificationAnimaliaClionaidaSpirastrellidae

Ridley, 1884

4D13131B-05FD-54D9-BF46-0E33B76611D3

Fig. 5

###### Diagnostic features.

**Figure 5.**
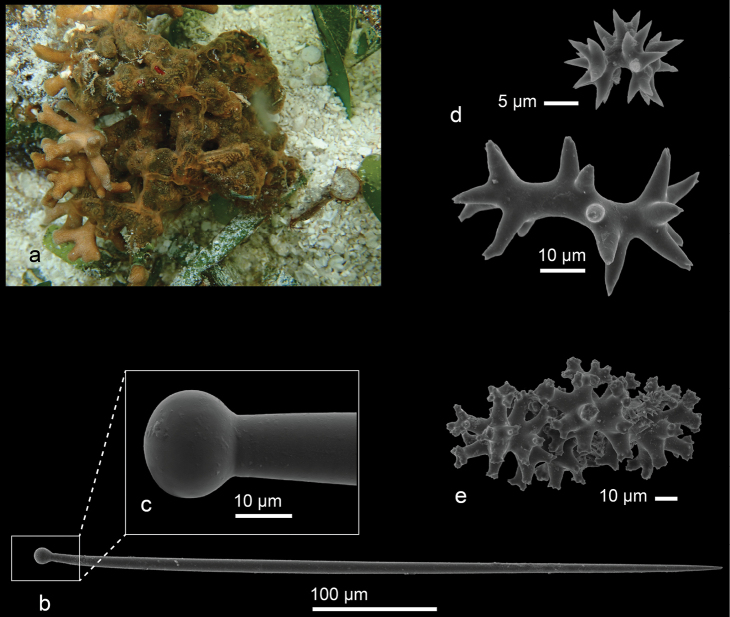
Spirastrellaaff.decumbens Ridley, 1884, overgrowing coral skeleton **a** habitus in situ (CEL007) from seagrass bed of Langkai Island, the Spermonde Archipelago (photograph by NJdeV) **b** SEM image of tylostyle with **c** close up of the head **d** spirasters **e** spirasters with ornamented rays.

A thin encrusting sponge with a soft texture and a smooth surface. The living specimens exhibit a salmon-pink or orangish color. The ectosome of the sponge contains numerous microscleres (spirasters), forming the characteristic tangential crust found in this genus. In the choanosome, the megascleres are irregularly arranged tylostyles with well-formed, usually spherical heads (Calcinai et al. 2006). Our specimen shows spirasters with ornamented rays (Fig. 5d) that are not mentioned in the Calcinai et al. (2006) report from Vietnam.

###### Distribution and ecology.

This species is present in the Australian region, New Caledonia, the Philippines, and Vietnam. In Indonesia is recorded from Ambon; this is a first record for the Spermonde Archipelago (Langkai Island; reef flat).

#### ﻿Order Haplosclerida Topsent, 1928

**Family Callyspongiidae de Laubenfels, 1936**

**Genus *Callyspongia* Duchassaing & Michelotti, 1864**

**Subgenus Cladochalina Schmidt, 1870**

##### ﻿ Callyspongia (Cladochalina) johannesthielei

Taxon classificationAnimaliaHaploscleridaCallyspongiidae

van Soest & Hooper, 2020

18E0B2A4-BE04-52D1-A26C-9585F68FC9E4

Fig. 6

###### Diagnostic features.

Lobate form and hard surface with numerous, raised, cone-shaped projections (pointed papillae). Several large oscula between ≈ 6–7 mm. Pink to red in living and pale yellow in alcohol. The skeleton is reticulate with a fiber tract. This species was described as *Spinosellaelegans* Thiele, 1899 (junior secondary homonym of Callyspongia (Cladochalina) elegans (von Lendenfeld, 1887)) as a large cup-shaped sponge, ≈ 30 cm high, hollow along its entire length, a pale brownish color when dry, and with very characteristic pointed papillae, often fused into a cluster of several, on the outer surface (Thiele 1899). The spicules of Thiele’s species were shown as rather thin, short-tipped amphioxeas that are 90–100 µm × 3–5 µm (van Soest et al. 2020).

**Figure 6.**
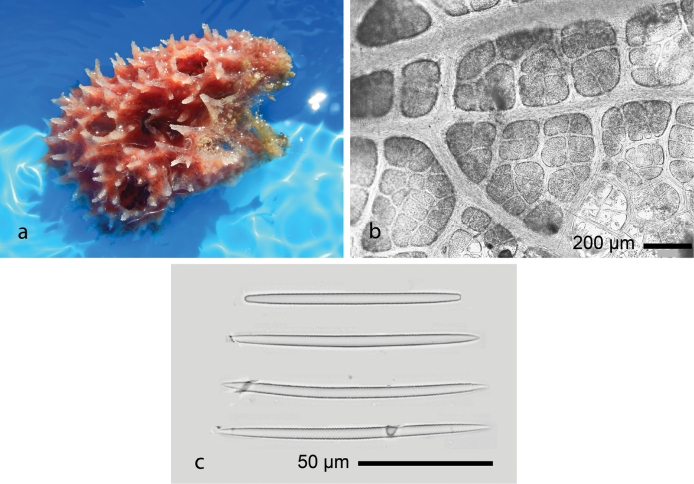
Callyspongia (Cladochalina) johannesthielei van Soest & Hooper, 2020 **a** habitus of fresh specimen (photograph by SAP) **b** skeleton **c** amphioxeas.

###### Distribution and ecology.

Kema Bay (1°23'N, 125°04'E), north Sulawesi (Thiele 1899); and north-west of Samalona Island, the Spermonde Archipelago; reef flat; attached on rock.

#### ﻿Family Chalinidae Gray, 1867

**Genus *Haliclona* Grant, 1841**

**Subgenus Gellius Gray, 1867**

##### ﻿ Haliclona (Gellius) cymaeformis

Taxon classificationAnimaliaHaploscleridaChalinidae

(Esper, 1806)

BACB59ED-79A3-5342-9C49-F80E75919C18

Fig. 7

###### Diagnostic features.

**Figure 7.**
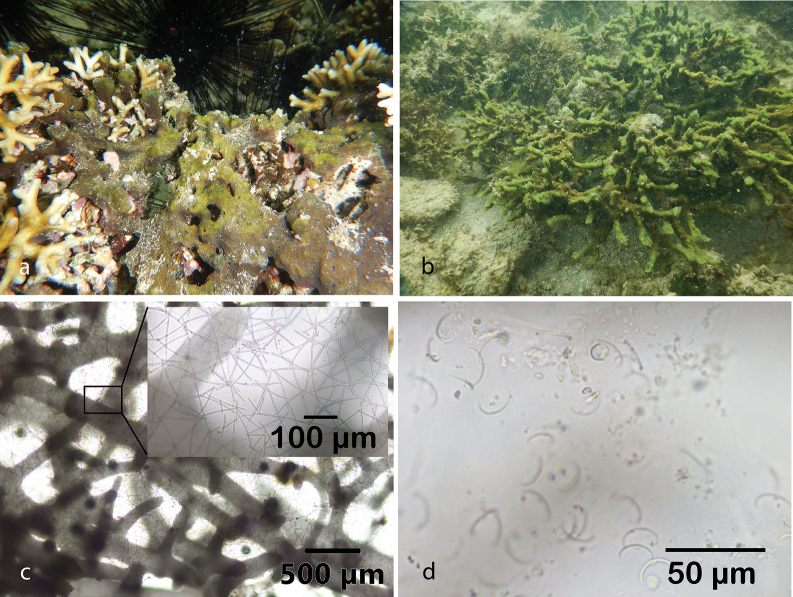
Haliclona (Gellius) cymaeformis (Esper, 1806) **a, b** habitus in situ at Samalona Island and Kayangan Island (respectively), the Spermonde Archipelago (all photographs by SAP) **c** LM images of tangential section showing Rhodophyta symbiont and unispicular tract (box) **d** sigmas.

The appearance is thickly encrusting to repent or arborescent (bushy). The specimen is hard and smooth on the surface, with a broad erect base with short branches. The color in life is dark greyish pink (dark purple) with desaturated dark green on the tips. After preservation, the color is pale pink to yellow. Ectosomal skeleton shows unispicular tract and covering the associated branching microalgae (Fig. 7c). Spicules are oxeas, 109–154 (129.7) × 2.3–5.2 (3.9) µm (*n* = 27), and microscleres are sigmas.

Haliclona (Gellius) cymaeformis (Esper, 1806) was abundant in turbid water near Makassar City. This species is known to be associated with the rhodophyte *Ceratodictyonspongiosum* Zanardini, 1878 (Azzini et al. 2007). Its morphological appearance is possibly similar to those of Halichondria (Halichondria) cartilaginea (Esper, 1797) and Callyspongia (Cladochalina) samarensis (Wilson, 1925).

###### Distribution and ecology.

This species has been recorded from marine karst lakes in Vietnam (Azzini et al. 2007), in shallow waters of the South China Sea (Huang et al. 2016; Lim et al. 2016), and in Taiwan (Li 2013), Andaman (Immanuel et al. 2015), India (George et al. 2020), across the Indonesian Archipelago (de Voogd and Cleary 2008), and north-west Australia (Fromont and Sampey 2014). Our samples were collected from a reef flat north-west of Samalona Island, overgrowing corals (*Seriatopora* sp. and *Acropora* sp.), also from Kayangan Island and Gusung Tallang; turbid reef environment.

#### ﻿Subgenus Reneira Schmidt, 1862

##### ﻿ Haliclona (Reniera) venusta

Taxon classificationAnimaliaHaploscleridaChalinidae

(Bowerbank, 1875)

CBC0555A-B8E5-5F3E-9323-F7A1036892D7

Fig. 8

###### Diagnostic features.

Specimen form tube, soft and delicate. Color yellowish in living material and yellow to pale white in alcohol. The skeleton forms an isotropic reticulation of a single line spicules. All spicules on this specimen are oxeas, 88–109 (95.2) × 4.3–6.5 (5.7) µm (*n* = 20).

###### Distribution and ecology.

The WPD checklist only lists four species of the subgenus Reniera recorded from marine ecoregions of Indonesia with two as doubtful species, Haliclona (Reniera) cinerea (Grant, 1826) (doubtful species), Haliclona (Reniera) fascigera (Hentschel, 1912), Haliclona (Reniera) infundibularis (Ridley & Dendy, 1887) (doubtful species), and Haliclona (Reniera) venusta (Bowerbank, 1875), but none of these species were registered in the Spermonde Archipelago (Putra et al. 2023). This report presents a new record of Haliclona (Reniera) venusta from the Spermonde Archipelago (Samalona Island; reef flat). Previously, this species has been only reported from Malacca Strait (Bowerbank 1875).

**Figure 8.**
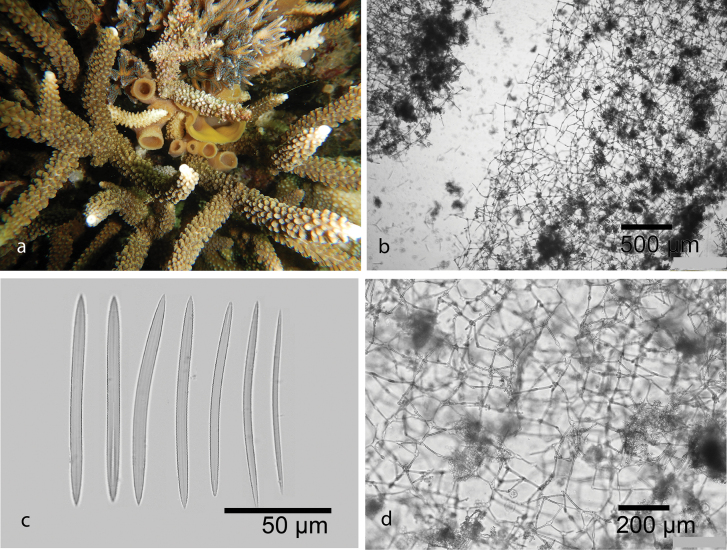
Haliclona (Reniera) venusta (Bowerbank, 1875) **a** habitus in situ at Samalona Island, the Spermonde Archipelago (photograph by SAP) **b** LM images of tangential section and spicules reticulation **c** oxeas **d** close up of spicules reticulation.

#### ﻿Subgenus Soestella de Weerdt, 2000

##### ﻿ Haliclona (Soestella) elegantia

Taxon classificationAnimaliaHaploscleridaChalinidae

(Bowerbank, 1875)

616D3B63-67EF-5186-8AE5-F96673DA990F

Fig. 9a, d, e

###### Diagnostic features.

Small specimen (l × w × h; 46 × 34 × 30 mm) and fragile, found growing in turbid water near the coastal city of Makassar. Massive shape with large oscula (3–4 mm in diameter). Color in life deep blue and pale white in alcohol. The choanosomal skeleton is paucispicular tracts. Spicules are oxeas, larger oxeas 163.9–196.2 (163.9) × 7–9.9 (8) µm (*n* = 20) and thin oxeas 92–156.1 (127.5) × 0.8–5.7 (3) µm (*n* = 26). Microscleres are sigmas. The subgenus Haliclona (Soestella) consists of 25 species, and only Haliclona (Soestella) elegantia is registered from the marine ecoregions of Indonesia (Putra et al. 2023). This species is poorly studied; in fact, we have found no studies after its original description. Bowerbank’s description did not include an illustration, but the specimen was described as of small appearance and small spicules (short and stout) with fragile and elegant uni-, bi-, and tri-spiculous reticulation on the dermal structure (Bowerbank 1875: 286).

**Figure 9.**
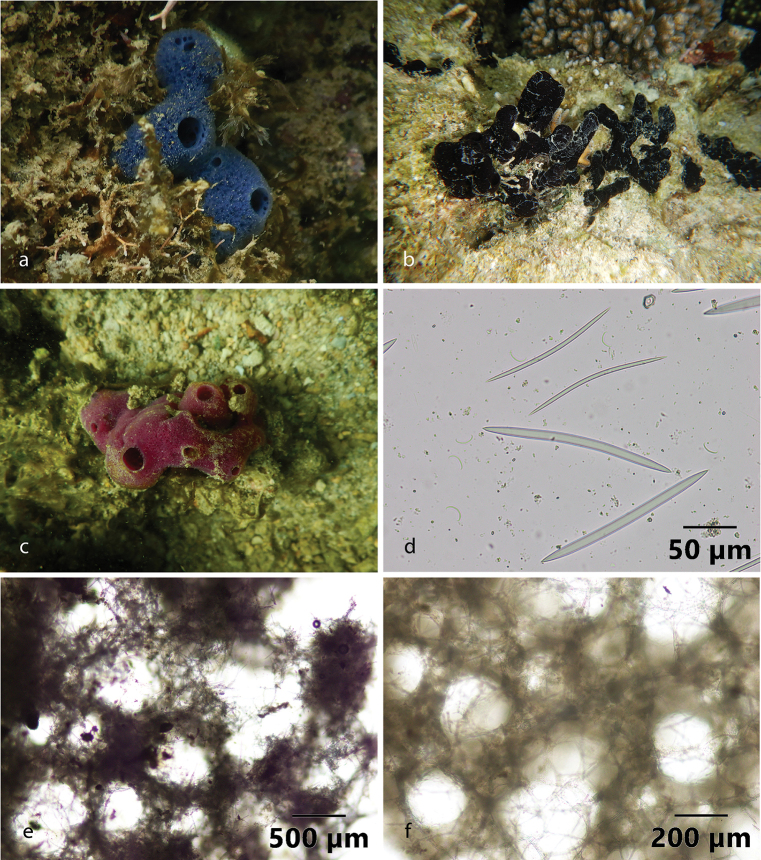
Habitus in situ **a**Haliclona (Soestella) elegantia (Bowerbank, 1875) at Kayangan Island, the Spermonde Archipelago **b**Haliclona (Soestella) sp. 1. at Samalona Island, the Spermonde Archipelago **c**Haliclona (Soestella) sp. 2. at Samalona Island, the Spermonde Archipelago (all photographs by SAP) **d** two sizes of oxeas and sigmas of Haliclona (Soestella) elegantia**e**Haliclona (Soestella) elegantia spicules reticulation **f**Haliclona (Soestella) sp. 2. spicule reticulation.

###### Distribution and ecology.

Previously recorded from Malacca Strait (Bowerbank 1875). This is the first record for the Spermonde Archipelago (at Kayangan Island, and Gusung Tallang Island; turbid environment).

##### ﻿ Haliclona (Soestella)

Taxon classificationAnimaliaHaploscleridaChalinidae

sp. 1

F14295D5-2944-5EA4-95CE-C7198C40A010

Fig. 9b

###### Diagnostic features.

The specimen is fragile and shapeless (amorphous), the surface is slick and smooth; the color in life is mostly black, also in alcohol. Oscula present with 1–3 mm diameter. The spicule arrangements are oxeas 101–162 (128.8) × 1.5–7 (4.9) µm (*n* = 21).

###### Distribution and ecology.

North-west Samalona Island, the Spermonde Archipelago; reef flat.

##### ﻿ Haliclona (Soestella)

Taxon classificationAnimaliaHaploscleridaChalinidae

sp. 2

D4778AB4-0232-529B-AE51-90B517EBC273

Fig. 9c, f

###### Diagnostic features.

Small specimen (l × w × h; 45 × 32 × 25 mm) with magenta color in life and pale white in alcohol. Massive shape with large osculum. Ectosomal skeleton shows multispicular fiber tracts. Spicules are oxeas, larger oxeas 102–130.9 (116.1) × 3.8–6.5 (5) µm (*n* = 24), and thin oxeas 78.4–114.4 (96.8) × 1.3–4.1 (2.5) µm (*n* = 20). Rounded meshes formed by the spicules characterized those species as belonging to the subgenus Soestella (de Weerdt 2000). However, due to differences in color and variation of the macro-morphology, it can be distinguished from Haliclona (Soestella) elegantia.

###### Distribution and ecology.

West Kayangan Island and Gusung Tallang Island, the Spermonde Archipelago; turbid environment.

#### ﻿Family Niphatidae van Soest, 1980

**Genus *Amphimedon* Duchassaing & Michelotti, 1864**

##### ﻿ Amphimedon paraviridis

Taxon classificationAnimaliaHaploscleridaNiphatidae

Fromont, 1993

E5E2A3A3-A0DE-55A9-AEDD-850B1A1B9BAA

Fig. 10

###### Diagnostic features.

Encrusting and soft, with small oscula and scattered ostia on the surface. Pale green in life and turning brown in alcohol. Skeleton isotropic reticulation arranged by oxeas 155–194 (173.5) × 5.9–8.1 (7.2) µm. *Amphimedonparaviridis* has similarities with *Amphimedonviridis* Duchassaing & Michelotti, 1864 from the Caribbean Sea. However, the holotype of *A.paraviridis* (from the Great Barrier Reef) has thicker spicules, a much greater spongin component, thicker fibers, and larger mesh spaces compared to *A.viridis* (Fromont 1993). Only three species of *Amphimedon* have been reported from the marine ecoregions of Indonesia (Putra et al. 2023), including *Amphimedonanastomosa*Calcinai et al., 2017, *Amphimedonzamboangae* (Lévi, 1961), and *Amphimedondenhartogi* de Voogd, 2003.

**Figure 10.**
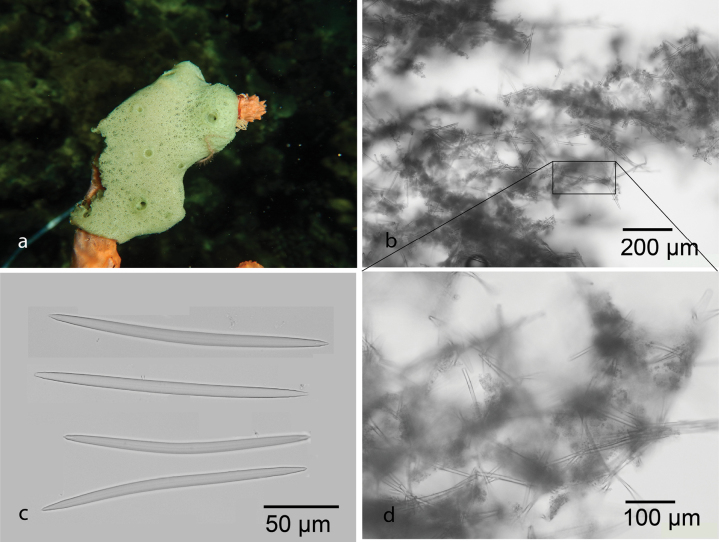
*Amphimedonparaviridis* Fromont, 1993 **a** habitus in situ over growing Clathria (Thalysias) reinwardti Vosmaer, 1880 at Samalona Island, the Spermonde Archipelago (photograph by SAP) **b** LM image of cross section of the skeleton **c** oxeas **d** Isotropic reticulation of oxeas.

###### Distribution and ecology.

Previously reported from Australia (Fromont 1993). This is first record of *Amphimedonparaviridis* from Samalona Island, the Spermonde Archipelago. Reef flat, overgrowing another sponge, Clathria (Thalysias) reinwardti Vosmaer, 1880.

#### ﻿Genus *Niphates* Duchassaing & Michelotti, 1864

##### ﻿ Niphates nitida

Taxon classificationAnimaliaHaploscleridaNiphatidae

Fromont, 1993

DDF8CC5C-60EF-531E-8D01-ADABD00DD639

Fig. 11

###### Diagnostic features.

Ramose repent sponge. Bluish green in life, pale white in alcohol. Oscula are small, 2–4 mm in diameter. Ectosomal shows reticulation fiber tract. Oxeas slightly curved, larger oxeas 120.3–171.3 (139.4) × 4.8–9.3 (6.1) µm (*n* = 22), thin oxeas 109.3–132.7 (121) × 2.4–5.3 (3.5) µm (*n* = 14). Microscleres are C-shaped sigmas. This specimen is identified as *Niphatesnitida* due to the reticulation fiber tract on the skeleton and the present of sigmas. Previously, only two species of *Niphates* recorded from Indonesia. *Niphateslaminaris*Calcinai et al., 2017 is characterized by a non-spiny, rather irregular, microconulose surface and a chaonosomal skeleton with primary and secondary reticulation fiber tracts, as well as numerous microscleres (Calcinai et al. 2017). *Niphatesolemda* (de Laubenfels, 1954) is a tubular sponge with small oxeas (de Laubenfels 1954). *Niphatesnitida* is a new record for Indonesia.

**Figure 11.**
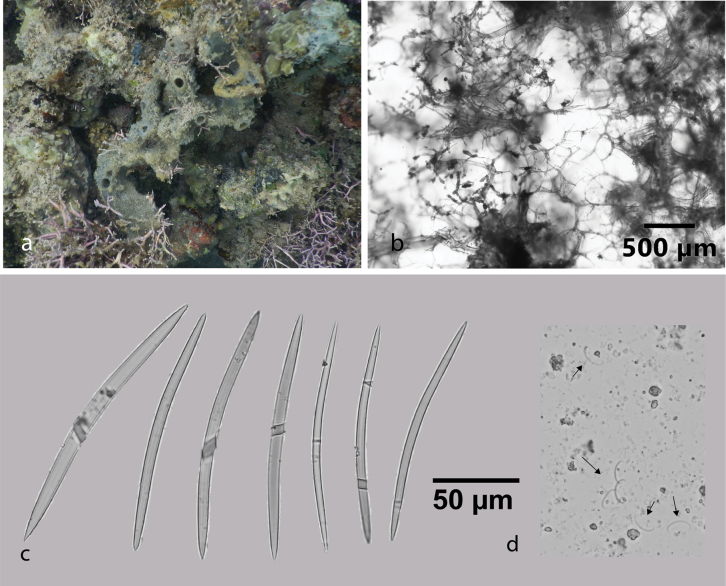
*Niphatesnitida* Fromont, 1993 **a** habitus in situ at Kayangan Island, the Spermonde Archipelago (photograph by SAP) **b** ectosmal skeleton **c** oxeas **d** sigmas

###### Distribution and ecology.

Previously was reported from Magnetic Island, Australia (Fromont 1993). This is first record for the Spermonde Archipelago (at Kayangan Island; turbid environment).

#### ﻿Family Petrosiidae van Soest, 1980

**Genus *Petrosia* Vosmaer, 1885**

**Subgenus Petrosia Vosmaer, 1885**

##### ﻿ Petrosia (Petrosia) hoeksemai

Taxon classificationAnimaliaHaploscleridaPetrosiidae

de Voogd & van Soest, 2002

5949209D-2A86-5D1D-8E5E-A89C06ABF5CF

Fig. 12

###### Diagnostic features.

The sponge is thick, massive, and encrusting with rugose surface. Color brown outside, cream inside, and turning blackish brown after preservation. Choanosomal skeleton shows pauci-multispicular spicule tracts. Three sizes of oxeas, primary oxeas 182.3–272.9 (219.6) × 10.8–19.2 (14.6) µm (*n* = 28), secondary oxeas 126.4–221.7 (173.6) × 6.7–11.4 (8.7) µm (*n* = 32), and tertiary oxeas 58–123.9 (83.1) × 5.6–10.5 (7.5) µm (*n* = 28).

Seven species of *Petrosia* have been reported from the Spermonde Archipelago, i.e., Petrosia (Petrosia) hoeksemai de Voogd & van Soest, 2002, Petrosia (Petrosia) alfiani de Voogd & van Soest, 2002, Petrosia (Petrosia) lignosa Wilson, 1925, Petrosia (Petrosia) nigricans Lindgren, 1897, Petrosia (Petrosia) plana Wilson, 1925, Petrosia (Strongylophora) cortica (Wilson, 1925), and Petrosia (Strongylophora) strongylata (Thiele, 1903). Two species were originally described from this area, Petrosia (Petrosia) alfiani and Petrosia (Petrosia) hoeksemai (de Voogd and van Soest 2002). Our specimen shows slightly bigger secondary and tertiary oxeas compare to the de Voogd & van Soest (2002) specimen. Comparison of spicules measurement between Indonesian *Petrosia* specimen are shown in Table 1.

**Figure 12.**
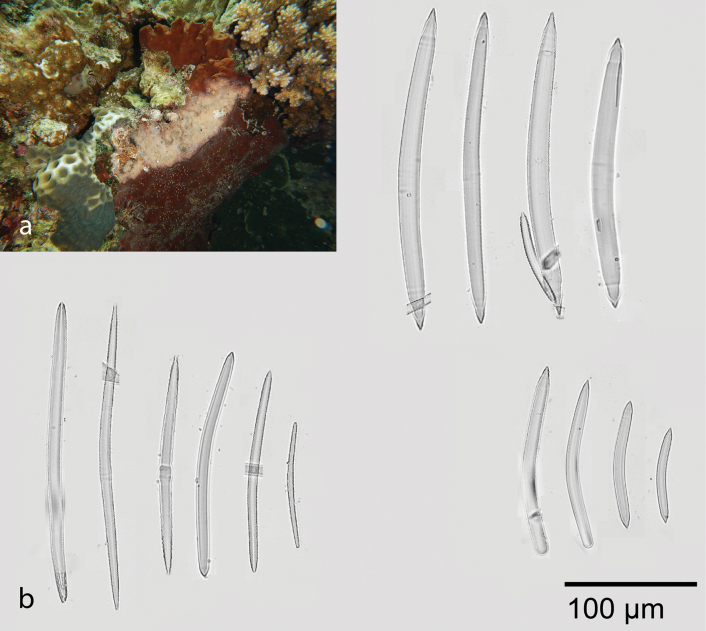
Petrosia (Petrosia) hoeksemai de Voogd & van Soest, 2002 **a** habitus in situ at Samalona Island, the Spermonde Archipelago (photograph by SAP) **b** three sizes of oxeas.

**Table 1.** Comparison of spicule measurements (µm) in specimens of Petrosia (Petrosia) and Petrosia (Strongylophora) from Indonesia.

Species	Oxeas/ Strongyles 1	Oxeas/ Strongyles 2	Oxeas/ Strongyles 3	Reference
Petrosia (Petrosia) hoeksemai	182.3–272.9 × 10.8–19.2	126.4–221.7 × 6.7–11.4	58–123.9 × 5.6–10.5	This study
Petrosia (Petrosia) hoeksemai	240–305 × 10–20	90–130 × 7–12	40–75 × 5–9	(de Voogd and van Soest 2002)
Petrosia (Petrosia) alfiani	183–253 × 10–15	106–153 × 7–14	60–70 × 6–7	(de Voogd and van Soest 2002)
Petrosia (Petrosia) lignosa	230–300 × 14–18	75–150 × 10–13	35–65 × 7–10	(de Voogd and van Soest 2002)
Petrosia (Petrosia) nigricans	240–305 × 8–16	120–188 × 9–10	57–85 × 5	(de Voogd and van Soest 2002)
Petrosia (Petrosia) plana	190–290 × 7–14	95–130 × 7–9.5	43–75 × 5–9	(de Voogd and van Soest 2002)
Petrosia (Strongylophora) cortica	300–360 × 11–14	80–200 × 11–14	21–50 × 3–9	(de Voogd and van Soest 2002)
Petrosia (Strongylophora) strongylata	326 × 18	95–145 × 10–12	44–60 × 8–12	(de Voogd and van Soest 2002)

###### Distribution and ecology.

Samalona Island, the Spermonde Archipelago, attached vertically; reef flat; also reported from north Sulawesi (de Voogd and van Soest 2002).

#### ﻿Order Poecilosclerida Topsent, 1928

**Family Coelosphaeridea Dendy, 1922**

**Genus *Lissodendoryx* Topsent, 1892**

**Subgenus Waldoshmittia de Laubenfels, 1936**

##### ﻿ Lissodendoryx (Waldoschmittia) schmidti

Taxon classificationAnimaliaPoeciloscleridaCoelosphaeridea

(Ridley, 1884)

1195CF22-CD2C-5010-AFCD-66F785083261

Fig. 13

###### Diagnostic features.

Ectosome is formed of tangentially arranged tylotes and ascending bundles in a plumose arrangement. Main skeleton is an irregular reticulation of oxeas, with triangular meshes of spicules. Microscleres are isochelas and sigmas (Hofman and van Soest 1995).

**Figure 13.**
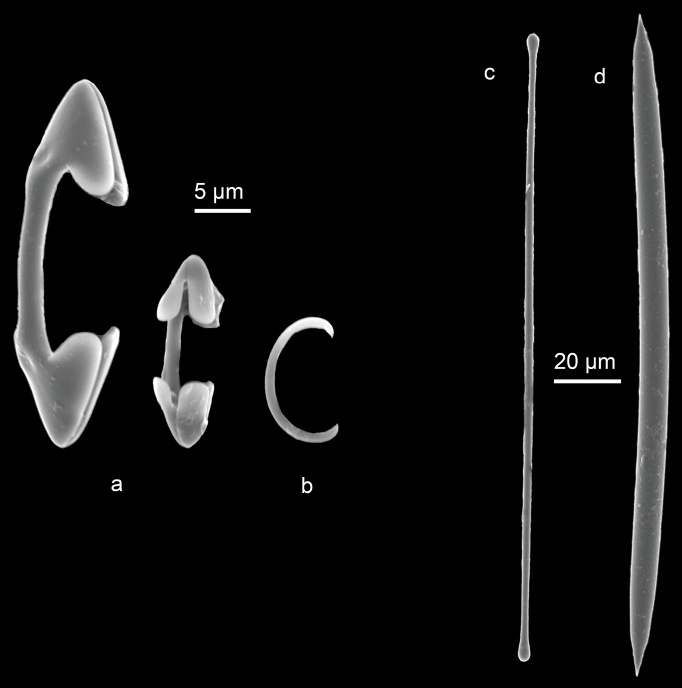
Lissodendoryx (Waldoschmittia) schmidti (Ridley, 1884) (CEL079) **a** SEM images of isochelae **b** sigma **c** tylote **d** oxea.

###### Distribution and ecology.

This species also known from mesophotic zone. Previously recorded from Cochin-China, East Africa, Hawaii, Red Sea, Seychelles, and South Australia. In parts of Indonesia it was recorded from Ternate, Banda Sea, Aru Island (Arafura Sea), Flores, Jedan Island, East Java, and Sumba (Hofman and van Soest 1995). Our specimen is the first record for the Spermonde Archipelago, Lumulumu Island; reef flat.

#### ﻿Family Iotrochotidae Dendy, 1922

**Genus *Iotrochota* Ridley, 1884**

##### ﻿ Iotrochota baculifera

Taxon classificationAnimaliaPoeciloscleridaIotrochotidae

Ridley, 1884

F073D4A3-3FFA-5BEC-A3F9-734601B94DD6

Fig. 14

###### Diagnostic features.

Black, thin, encrusting with rough surface, and boring. Choanosomal skeleton show multispicular reticulation. Spicule arrangements are styles 157.9–212.5 (191.7) × 7.4–15.9 (11.4) µm (*n* = 25), strongyles 248–287.6 (266.6) × 3.6–7.8 (6.7) µm (*n* = 25), with microsclere birotulate chelae, 13.9–17.3 (15.4) µm (*n* = 21). *Iotrochotabaculifera* has similar coloration with *Iotrochotapurpurea* (Bowerbank, 1875) and *Iotrochotanigra* (Baer, 1906). Table 2 shows the comparison of the spicule measurements of these species.

**Figure 14.**
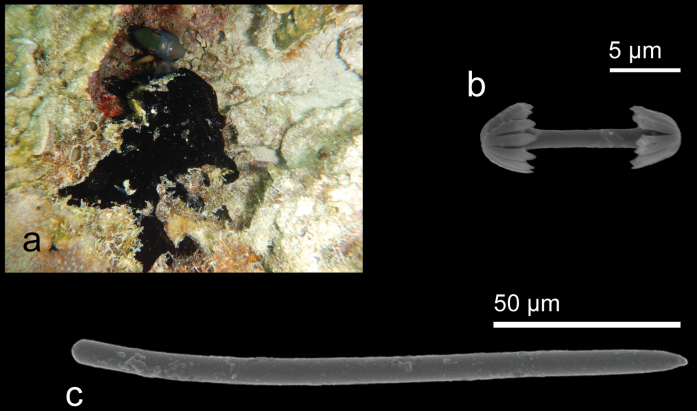
*Iotrochotabaculifera* Ridley, 1884 **a** habitus in situ at Samalona Island, the Spermonde Archipelago (photograph by SAP) **b** birotulate chelae **c** styles.

**Table 2.** Comparison of spicule measurements (µm) in specimens of *Iotrochotabaculifera*, *Iotrochotapurpurea*, and *Iotrochotanigra*.

Species	Styles	Strongyles	Birotulates	Reference
*Iotrochotabaculifera*	157.9–212.5 (191.7) × 7.4–15.9 (11.4)	248–287.6 (266.6) × 3.6–7.8 (6.7)	13.9–17.3	This study
*Iotrochotabaculifera*	200 × 9.5–12.7	220–280 × 6.3	16	(Ridley 1884)
*Iotrochotabaculifera*	125–180 × 5.5–7.5	225–255 × 3.5–5	13–16.5	(Bergquist 1965)
*Iotrochotabaculifera*	168–189 (175) × 4–8 (6)	201–243 (225) × 4–6 (4)	12	(Thomas 1973)
*Iotrochotabaculifera*	145–170 (160) × 5–8.7 (7.5)	205–230 (220.9) × 2.5–5 (4)	12	(Núñez Pons et al. 2017)
*Iotrochotapurpurea*	146–180(163) × 4–8(5)	-	16	(Thomas 1973)
*Iotrochotapurpurea*	168 × 8	-	-	(Thomas 1991)
*Iotrochotanigra*	170 × 6	-	-	(Baer 1906)
*Iotrochotanigra*	230–269 (251) × 5 (5)	163–193(184) × 7(7)	17(17)	(Samaai et al. 2019)

###### Distribution and ecology.

Widespread from the Western Indian Ocean to Hawaii (Núñez Pons et al. 2017). Only two species of *Iotrochota* have been recorded from Spermonde Archipelago, *Iotrochotapurpurea* and *Iotrochotabaculifera* Ridley, 1884 (de Voogd 2005). Our specimen was found in the north-west of Samalona Island, the Spermonde Archipelago; reef flat.

#### ﻿Family Microcionidae Carter, 1875

**Genus *Clathria* Schmidt, 1862**

**Subgenus Thalysias Duchassaing & Michelotti, 1864**

##### ﻿ Clathria (Thalysias) reinwardti

Taxon classificationAnimaliaPoeciloscleridaMicrocionidae

Vosmaer, 1880

08ABD178-565D-5FDF-89A5-0527F8B51B4C

Fig. 15

###### Diagnostic features.

Arborescent, simple massive, and very repent appearance with many small oscula. Bright to dark orange in living material, and brown in alcohol. Reticulate skeleton with two class sizes of styles and echinating acanthostyles. Principal styles slightly curved with strongylote point, 151–312 (205.5) × 5.3–10.85 (7.4) µm (*n* = 28), auxiliary styles straight and slightly curved, 72–163 (106.5) × 1.5–4.7 (3.4) µm (*n* = 37), and echinating acanthostyles with short, rounded point and dense spines on point and base, 51.9–81.5 (67.1) × 6.2–8.7 (7.3) µm (*n* = 31). This species can be differentiated from other similar *Thalysias* by its characteristic acanthostyle morphology, growth form, and the size and geometry of its toxas, including ectosomal-subectosomal features (Hooper 1996). Hooper’s (1996) specimen shows microscleres as palmate isochelae in two size classes and oxhorn toxas.

**Figure 15.**
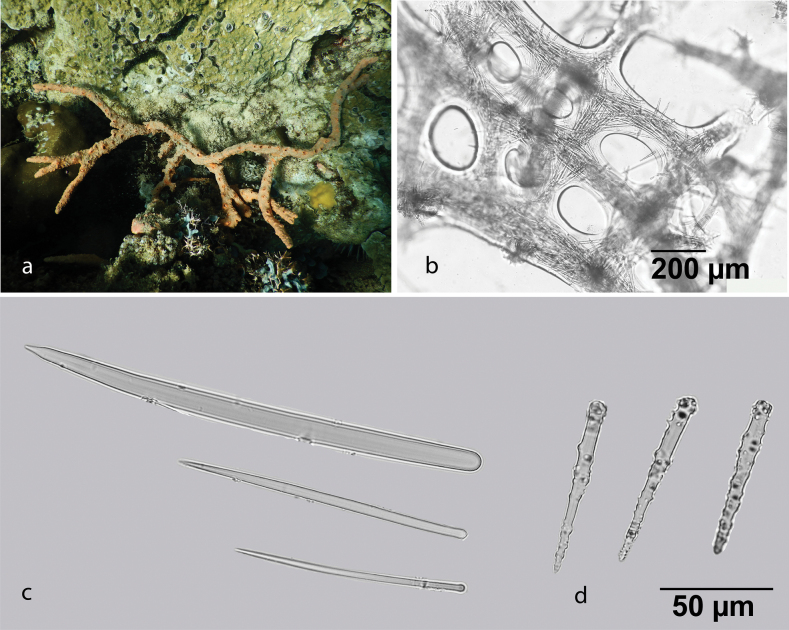
Clathria (Thalysias) reinwardti Vosmaer, 1880 **a** habitus in situ at Samalona Island, the Spermonde Archipelago (photograph by SAP) **b** longitudinal section of the skeleton **c** styles **d** acanthostyles.

###### Distribution and ecology.

Central Indian Ocean (Thomas 1986), Indo-Pacific (van Soest 1990; Lim et al. 2016), and Australia (Hooper 1996). Commonly found in coral rubble or dead coral and hard substrates. Our specimen was found in the Spermonde Archipelago, the north-west of Samalona Island; reef flat and Gusung Tallang; turbid reef.

#### ﻿Order Scopalinida Morrow & Cárdenas, 2015

**Family Scopalinidae Morrow et al., 2012**

**Genus *Stylissa* Hallmann, 1914**

##### ﻿ Stylissa massa

Taxon classificationAnimaliaScopalinidaScopalinidae

(Carter, 1887)

96492775-4740-5533-A439-EF767515F21B

Fig. 16

###### Diagnostic features.

Massive, soft, and friable with rough surface and medium-sized oscula appear on top of the ridge. Yellow-orange in life and brown in alcohol. Spicules arrangements are of styles and strongyles.

###### Distribution and ecology.

*Stylissamassa* is widely distributed in the Indo-Pacific (Erpenbeck et al. 2017). Since *Stylissamassa* is known to be widespread, and recent studies using molecular techniques show the probability of distinct cryptic lineages of this species in the Indo-Pacific (Erpenbeck et al. 2017). Our specimen was collected from the Spermonde Archipelago, south-west of Samalona Island; reef flat, attached to rubble and dead coral skeletons.

**Figure 16.**
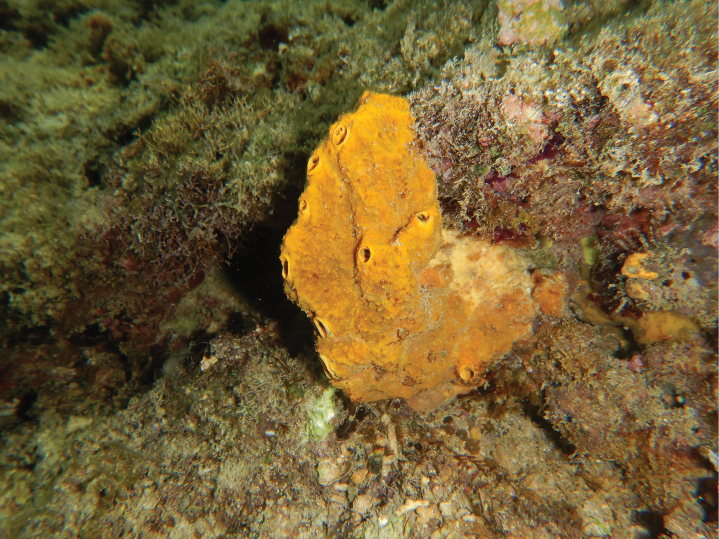
Habitus in situ of *Stylissamassa* (Carter, 1887) at Samalona Island, the Spermonde Archipelago (photograph by SAP).

#### ﻿Order Suberitida Chombard & Boury-Esnault, 1999

**Family Halichondriidae Gray, 1867**

**Genus *Halichondria* Fleming, 1828**

**Subgenus Halichondria Fleming, 1828**

##### ﻿ Halichondria (Halichondria) cartilaginea

Taxon classificationAnimaliaSuberitidaHalichondriidae

(Esper, 1797)

3AD7F13E-34A9-5044-B113-8D9C3D4A2408

Fig. 17

###### Description.

Massive creeping growth form with upright branches (branching). These branches are irregular and form mats covering the substrate. Color bright green, flexible/cartilaginous. This species lives in association with Chlorophyta*Cladophoropsisvaucheriiformis* (Areschoug) Papenfuss, 1958 (van Soest 1990). Spicules are only oxeas, 125.46–252.45 (191.40) × 4.03–7.40 (5.37) µm.

**Figure 17.**
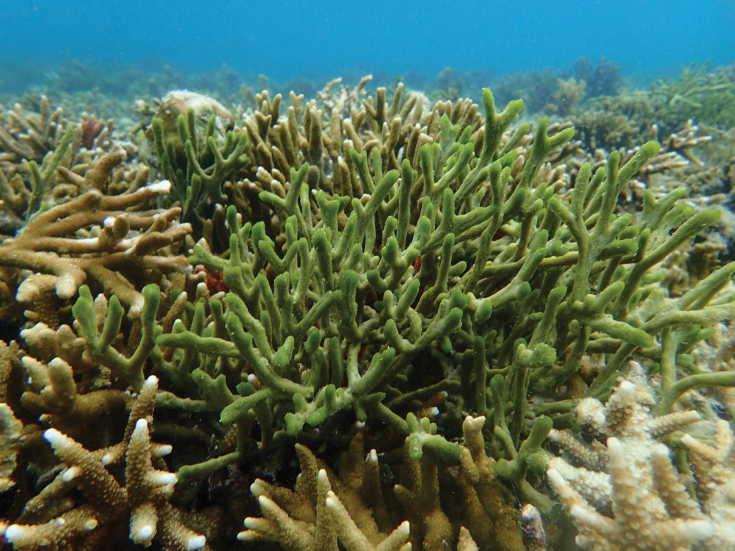
Habitus in situ of Halichondria (Halichondria) cartilaginea (Esper, 1797) (CEL025) (photograph by NJdeV).

###### Distribution and ecology.

Currently this species is recorded from China, Vietnam, Malacca Strait, Banda Sea, and East African Coral Coast. According to the WPD checklist (de Voogd et al. 2024), this is the first record from the Spermonde Archipelago, Badi Island; reef flat.

#### ﻿Genus *Topsentia* Berg, 1899

##### ﻿ Topsentia indica

Taxon classificationAnimaliaSuberitidaHalichondriidae

Hentschel, 1912

AFBA3B5A-A5F1-5253-A28E-F2ABE7F53C00

Fig. 18

###### Description.

Only two species of *Topsentia* are distributed in Indonesia, i.e., *Topsentiadura* (Lindgren, 1897) and *Topsentiaindica* Hentschel, 1912. *Topsentiadura* had further illustrations and spicule measurements provided by a previous study (Alvarez and Hooper 2011). These species are massive, of hard consistency with skeletons made of a confused mass of oxeas of similar dimensions, not clearly differentiated into size classes. Our specimen shows similar characteristics with the specimen of Hentschel (1912).

**Figure 18.**
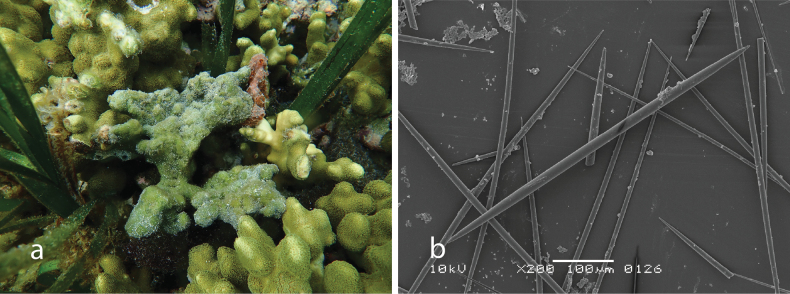
*Topsentiaindica* Hentschel, 1912 **a** habitus in situ at Langkai Island, the Spermonde Archipelago (photograph by NJdeV) **b** SEM images of the spicules.

###### Distribution and ecology.

Previously recorded from Aru Islands (Hentschel 1912). This is first record from the Spermonde Archipelago, Langkai Island; reef flat.

#### ﻿Family Suberitidae Schmidt, 1870

**Genus *Suberites* Nardo, 1833**

##### ﻿ Suberites

Taxon classificationAnimaliaSuberitidaSuberitidae

sp.

86339C4C-12EE-590A-8AAC-64ED7B5E51AB

Fig. 19

###### Diagnostic features.

Ficiform (fig-shaped) with orange (almost red) color and fragile. Oscula found on top of the fig-like shape. Aquiferous network can be seen from ectosomal skeleton of living specimen, small ostia also visible. Spicules are tylostyles (total length × width) 204.3–324.5 (278.4) × 3.5–8.6 (5.5) µm (*n* = 31). Tylostyle heads are oval with an indistinct neck (head length × head width × neck width) 8.8–15.9 (12.3) × 4.2–8.8 (6) × 3.2–8 (4.5) µm (*n* = 25).

**Figure 19.**
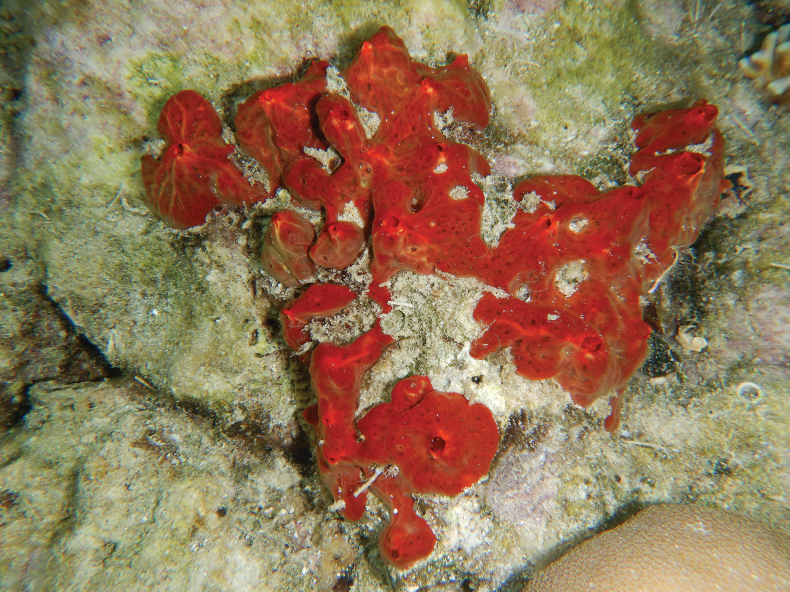
Habitus in situ of *Suberites* sp. (photograph by SAP).

###### Distribution and ecology.

Only three *Suberites* species have been recorded from Indonesia, *Suberitesradiatus* Kieschnick, 1896, *Suberitesdiversicolor* Becking & Lim, 2009, and the deep-sea *Suberitesperfectus* Ridley & Dendy, 1886 (Becking and Lim 2009; Putra et al. 2023). North-west of Samalona Island, the Spermonde Archipelago; reef flat, scattered across shallow water area, growth on rock, plastic PVC, and sometimes competing with Scleractinia.

#### ﻿Genus *Terpios* Duchassaing & Michelotti, 1864

##### ﻿ Terpios hoshinota

Taxon classificationAnimaliaSuberitidaSuberitidae

Rützler & Muzik, 1993

E88FCA49-28C3-5849-B5C0-A0A912D67B56

Fig. 20

###### Diagnostic features.

Thin (< 1 mm thick), encrusting, and excavating form overgrowing host coral skeletons (*Acropora* spp.). Dark grey to black, sometime pale grey in the upper surface. Original description of *Terpioshoshinota* show spicules as only tylostyles (Rützler and Muzik 1993). In this study, spicule arrangements are tylostyles (total length × width) 132.9–252 (206.9) × 2.6–7.8 (4.4) µm (*n* = 52), and variation of heads (head length × head width × neck width) 3.7–7.4 (5.4) × 4.8–9 (6.5) × 1.8–5 (3.3) µm (*n* = 27). Spicule dimension measurements are shown on Table 3. The morphology of *Terpioshoshinota* is similar to *Terpiosgranulosus* Bergquist, 1967 from Hawaiian reefs. The difference is that this species is greyish brown, has lobe-headed tylostyles, and has a cyanobacterial symbiont (Rützler and Muzik 1993). This species known as a coral-killing sponge, but a recent study shows *Terpioshoshinota* could also grow on glass slides, plastic sheets, and rubber tyres. The competitive interaction with the coral host is only for substrate rather than food or nutrients (Syue et al. 2021).

**Figure 20.**
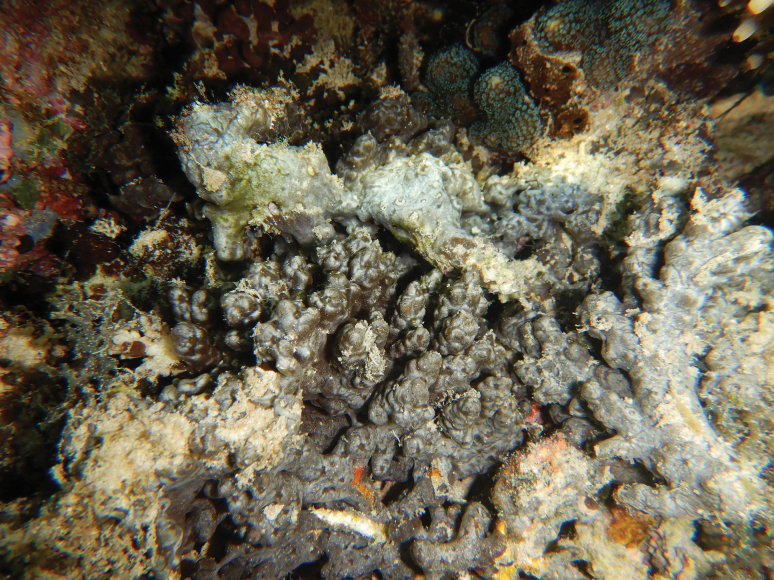
Habitus in situ of *Terpioshoshinota* Rützler & Muzik, 1993 (photograph by SAP).

**Table 3.** Spicule (tylostyles) dimensions (µm) for *Terpioshoshinota*.

Total length	Shaft width	Neck width	Head width	Head length	Reference
132.9–252 (206.9)	2.6–7.8 (4.4)	1.8–5 (3.3)	4.8–9 (6.5)	3.7–7.4 (5.4)	This study
180–290 (251.6)	3–4 (3.5)	2–3 (2.7)	5.5–7 (6.1)	4.5–6 (5.2)	(Rützler and Muzik 1993)

###### Distribution and ecology.

This widespread species has been recorded from the Indian Ocean, north-western Pacific, and Australia (Fromont et al. 2019). *Terpioshoshinota* was originally described from the Ryukyu Archipelago, Japan (north-west Pacific). Our specimen was found from north-west of Samalona Island, the Spermonde Archipelago; reef flat, overgrowing branching *Acropora* sp.

#### ﻿Order Tetractinellida Marshall, 1876

**Family Ancorinidae Schmidt, 1870**

**Genus *Ecionemia* Bowerbank, 1862**

##### ﻿ Ecionemia acervus

Taxon classificationAnimaliaTetractinellidaAncorinidae

Bowerbank, 1862

A850526F-7592-54A3-A344-215DA21D3F57

Fig. 21

###### Description.

Massive or thickly encrusting sponges without a distinct cortex. Megascleres are triaenes of different types and large oxeas. Microscleres include spiny microrhabds in addition to euasters. Microrhabds usually form a dermal layer (Uriz 2002).

**Figure 21.**
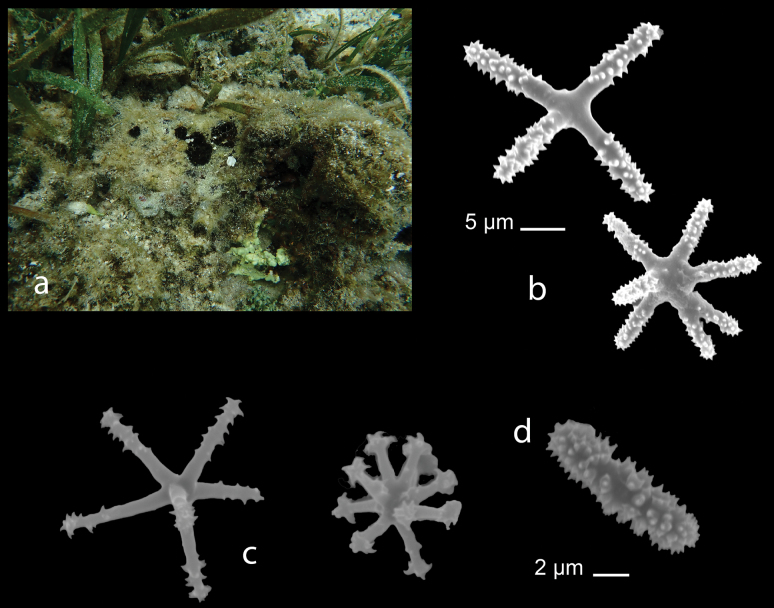
*Ecionemiaacervus* Bowerbank, 1862 **a** habitus in situ at Langkai Island, the Spermonde Archipelago (photograph by NJdeV) (CEL016), and SEM images of spicules, **b, c** somal chiasters/ strongylasters **d** cortical rough microrhabds/ microstrongyle.

###### Distribution and ecology.

Indo-Pacific, Australia, New Zealand. This species is common in the Indo-Pacific (Uriz 2002). Our specimen was collected from Langkai Island, the Spermonde Archipelago; reef flat.

#### ﻿Family Geodiidae Gray, 1867

**Genus *Geodia* Lamarck, 1815**

##### ﻿ Geodia

Taxon classificationAnimaliaTetractinellidaGeodiidae

sp.

9F987FDE-E84D-5F0F-A0E0-D01E1D25E82F

Fig. 22

###### Diagnostic features.

Twelve species of *Geodia* spp. were described from Indonesia (Putra et al. 2023; de Voogd et al. 2024). Our specimen has oxeas 1079.43–1820.54 (1507.20) × 18.17–33.67 (25.21) µm (*n* = 11), sterrasters widths 49.05–77.40 (59.98) µm (*n* = 20), dichotriaene, anatriaene, protriaene, strongylasters, and oxyasters. Further analysis is needed to examine and provide a name for this specimen.

**Figure 22.**
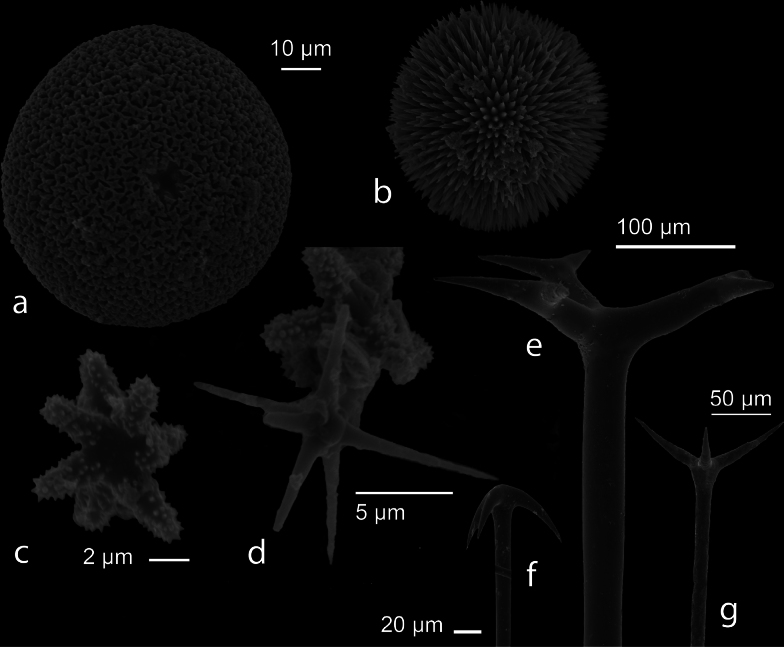
SEM images of spicules of *Geodia* sp. (CEL174) **a, b** sterrasters (**b** developmental stage) **c** strongylaster **d** oxyaster attached to strongylasters **e** dichotriaene **f** anatriaene **g** protriaene.

###### Distribution and ecology.

This group is distributed across Indonesia, i.e., Halmahera, Arafura Sea, Southern Java, Sunda Shelf/Java Sea, Banda Sea, Palawan/North Borneo (Sollas 1888; Kieschnick 1896; Topsent 1897; Lindgren 1898; Thiele 1900; von Lendenfeld 1903; Hentschel 1912; Wilson 1925; van Soest et al. 2020). Our specimen was collected from Barangbaringan Island, the Spermonde Archipelago; turbid environment.

#### ﻿Family Tetillidae Sollas, 1886

**Genus *Paratetilla* Dendy, 1905**

##### ﻿ Paratetilla bacca

Taxon classificationAnimaliaTetractinellidaTetillidae

(Selenka, 1867)

13BC455A-EB47-5BFE-A9D0-AAEED50EB32A

Fig. 23

###### Diagnostic features.

Globular sponges, specimen ≈ 64 × 47 mm (l × w) in diameter. Porocalices are abundant as circular to oval apertures. Color generally bright yellow when alive with brownish appearance in situ due to algal and sediment cover. Skeleton composed of oxea and triaenes radiating from a central core. Megascleres are oxeas, anatriaenes, and calthrops-like. Microscleres are sigmaspires, C- to S-shaped. A complete redescription of *P.bacca* was provided recently (Santodomingo and Becking 2018). This species had a considerable variation in spicules sizes in the different localities as well as significant intra-specific variation. This variation could be a response to different environmental conditions, a consequence of genetic selection, or synergistic between ecological and genetic factors.

**Figure 23.**
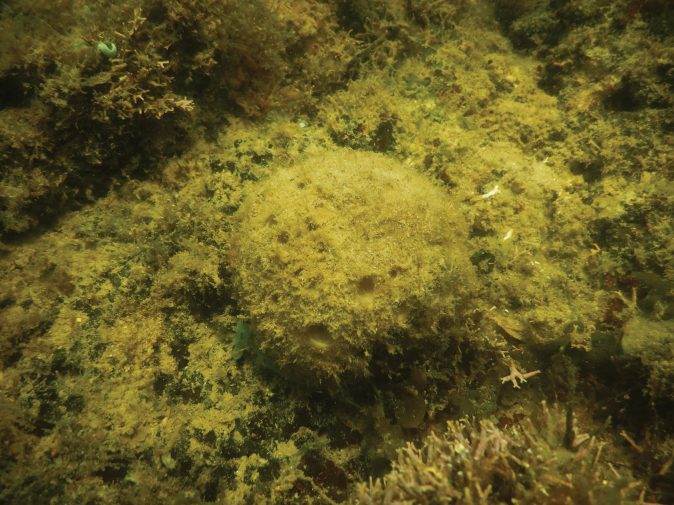
Habitus in situ of *Paratetillabacca* (Selenka, 1867) at Gusung Tallang, the Spermonde Archipelago (photograph by SAP).

###### Distribution and ecology.

Seychelles Islands (Thomas 1973), south-west Madagascar (Vacelet et al. 1976), Zanzibar (Pulitzer-Finali 1993), Thailand (Putchakarn 2007), Singapore (Lim et al. 2012), Philippines (Longakit et al. 2005), and Indonesia (Santodomingo and Becking 2018). Our specimen was collected from Gusung Tallang Island, the Spermonde Archipelago; turbid environment.

#### ﻿Subclass Keratosa Grant, 1861

**Order Dictyoceratida Minchin, 1900**

**Family Dysideidae Gray, 1867**

**Genus *Lamellodysidea* Cook & Bergquist, 2002**

##### ﻿ Lamellodysidea herbacea

Taxon classificationAnimaliaDictyoceratidaDysideidae

(Keller, 1889)

4BEB5C7F-FCD2-5788-BEC3-55B7AC5AF391

Fig. 24

###### Diagnostic features.

Live specimen found was white to pale green in color, and grey after preservation. This species habitus is soft, fragile, slick, thin (< 1 cm thick), and has an encrusting basal plate with a complex labyrinthine wall-like pattern. Skeleton structure forming interconnected reticulate fibers with several adjacent spicules. Various of microsymbionts (cyanobacteria) are found inhabiting it. Currently there only two species of *Lamellodysidea*, *Lamellodysideaherbacea* (Keller, 1889) and *Lamellodysideachlorea* (de Laubenfels, 1954), both confused with each other. *Lamellodysideaherbacea* is known to be common in the sub-intertidal zone of the coral reef, which is exposed to sunlight (Putchakarn 2007). Molecular analysis shows *Lamellodysideaherbacea* is a diverse group and consists of several distinct lineages of the alleged single species, and has probably been misidentified in the past with undescribed lineages due to superficial resemblances (Erpenbeck et al. 2012).

**Figure 24.**
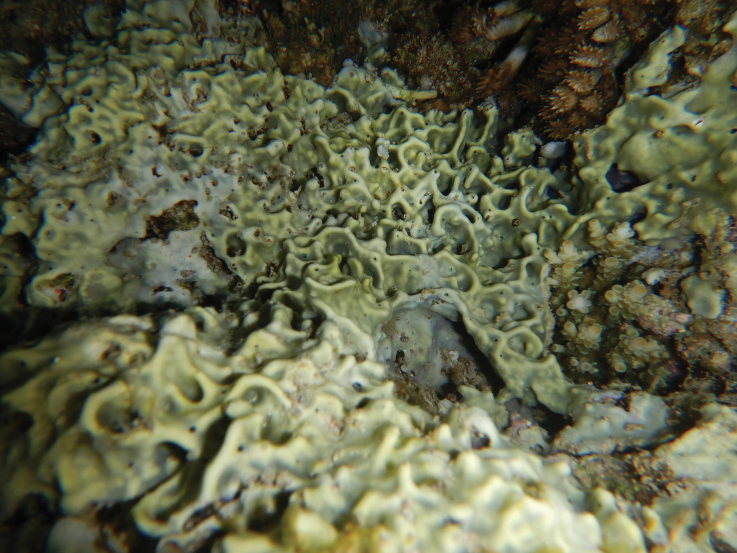
Habitus in situ of *Lamellodysideaherbacea* (Keller, 1889) at Samalona Island, the Spermonde Archipelago (photograph by SAP).

###### Distribution and ecology.

Our specimen was collected from Samalona Island, the Spermonde Archipelago; reef flat. This species was previously recorded from the Red Sea (Row 1911), India (George et al. 2020), Thailand (Putchakarn 2007), the Spermonde Archipelago (de Voogd et al. 2006), and the Great Barrier Reef (Hooper 2008).

#### ﻿Family Irciniidae Gray, 1867

**Genus *Ircinia* Nardo, 1833**

##### ﻿ Ircinia schulzei

Taxon classificationAnimaliaDictyoceratidaIrciniidae

(Dendy, 1905)

65A51917-C90B-55C6-9221-6136633AA2EC

Fig. 25

###### Diagnostic features.

Specimen attached to hard substrate, cylindrical with irregular short or club-shaped branches and rugose surface. Color in life is pale green and pale white in alcohol. Small oscula are found in every branch, sometimes on the tip. Skeleton is laminated fiber. Irciniidae are massive, or occasionally encrusting, sponges that display a wide range of forms, e.g., caliculate, lamelliform, lobate, and digitate. The species of *Ircinia* are pithed and laminated with primary and secondary fibers (de C. Cook and Bergquist 1999).

**Figure 25.**
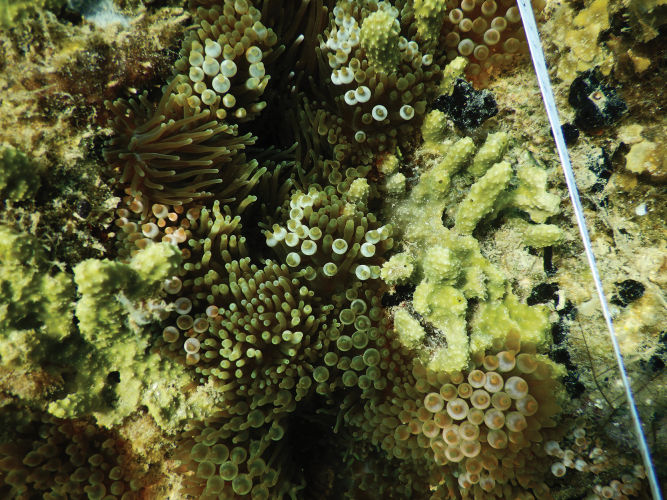
Habitus in situ of *Irciniaschulzei* (Dendy, 1905) at Samalona Island, the Spermonde Archipelago (photograph by SAP).

###### Distribution and ecology.

*Irciniaschulzei* was first described from Ceylon (Sri Lanka today; Dendy 1905). A previous record from Papua New Guinea (Pulitzer-Finali and Pronzato 1999) and this new record in the Spermonde Archipelago shows that the species could be widespread in the Indo-Pacific region. Our specimen was found living between an anemone and other sponges on top of a rock in the reef flat of north-west of Samalona Island, the Spermonde Archipelago.

#### ﻿Family Thorectidae Bergquist, 1978

**Genus *Phyllospongia* Ehlers, 1870**

##### ﻿ Phyllospongia foliascens

Taxon classificationAnimaliaDictyoceratidaThorectidae

(Pallas, 1766)

B1CE88D9-4757-5D44-9C41-5D964A1ED94C

Fig. 26

###### Diagnostic features.

Specimen form is foliaceous and irregular flabellate branches, pale white color in life and when preserved, < 0.5 mm thick. Numerous small oscula (< 1 mm) scattered in the surface. Skeleton consists of interconnected reticulate fibers. This species was recently transferred from the genus *Carteriospongia* Hyatt, 1877 due to molecular phylogenetic analysis showing *Carteriospongiafoliascens* as a clade member of *Phyllospongiabergquistae* Abdul Wahab & Fromont, 2020. The original diagnosis describing a verrucose surface is characteristic for *Phyllospongiafoliascens*, but with a fine and meandering surface patterning for *Phyllospongiabergquistae* (Bergquist et al. 1988; Abdul Wahab et al. 2021). *Phyllospongiafoliascens* is a phototrophic species that mainly relies on symbiotic cyanobacteria for its nutrient cycle. This species is also able to endure high energy environments (Cleary et al. 2005).

**Figure 26.**
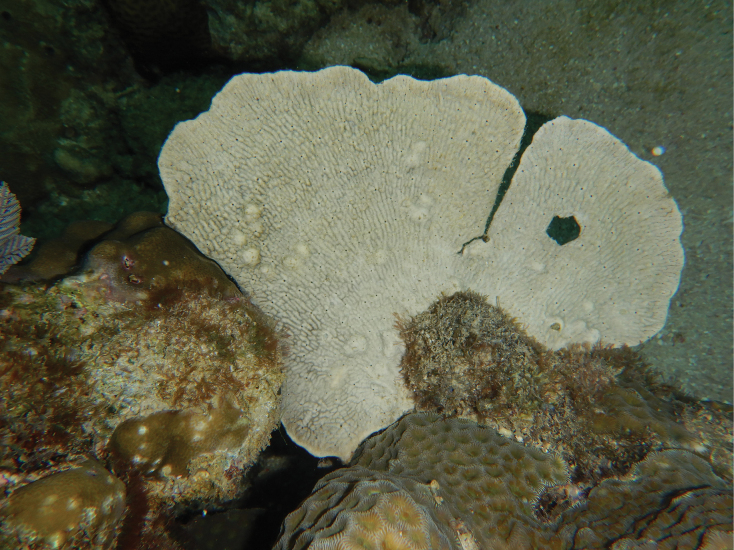
Habitus in situ of *Phyllospongiafoliascens* (Pallas, 1766) at Samalona Island, the Spermonde Archipelago (photograph by SAP).

###### Distribution and ecology.

Numerous individuals were found during the survey. *Phyllospongiafoliascens* is widely distributed and has a high density in the Spermonde Archipelago (de Voogd et al. 2006). Our specimen was found at south-west of Samalona Island, reef flat; Gusung Tallang, turbid reef. This species has been recorded from shallow waters of the Red Sea, Indian Ocean, Australia, and Fiji (Abdul Wahab et al. 2021).

## ﻿Discussion

Twenty-seven species of marine sponges (Class Calcarea and Class Demospongiae) have been identified in the littoral area of the Spermonde Archipelago, Indonesia. The Order Haplosclerida, with nine species, dominates this type of habitat. According to the WPD checklist (de Voogd et al. 2024), some of the sponges found here, such as *Clathrinarodriguesensis*, *Amphimedonparaviridis*, *Niphatesnitida*, and *Irciniaschulzei*, are newly recorded for Indonesia. Several others are new records for the Sulawesi Sea/Makassar Strait marine ecoregion, including *Janusyatubuloreticulosa*, *Leucaltisnodusgordii*, Spirastrellaaff.decumbens, Haliclona (Reniera) venusta, Haliclona (Soestella) elegantia, Lissodendoryx (Waldoschmittia) schmidti, Halichondria (Halichondria) cartilaginea, and *Topsentiaindica* (Suppl. material 2). Four species potentially new to science are also preliminarily described; further examination, including molecular analysis, is needed to accurately describe all the species.

In relation to extreme habitats, several species such as *Phyllospongiafoliascens*, *Stylissamassa*, Clathria (Thalysias) reinwardti, and Haliclona (Gellius) cymaeformis are frequently found in this habitat (Suppl. material 3). For instance, the foliose sponge *Phyllospongiafoliascens* as well as Haliclona (Gellius) cymaeformis were very abundant in the turbid reef near Makassar city, e.g., Kayangan Island, Gusung Tallang, and Samalona Island (SAP pers. obs. 2020). This habitat is unusual for phototropic species. Studies in other areas (i.e., north-west Java, the Great Barrier Reef) have shown that they are typically found in oligotrophic environments, characterized by low concentrations of organic nutrients (Wilkinson 1988; de Voogd and Cleary 2008). Conversely, several variables could be influencing the presence of these species in this unique environment. This could also be altered by algal symbionts that provide all the required carbon through photosynthesis, and the nitrogen from heterotrophic sources such as ultra-plankton (Davy et al. 2002; Pile et al. 2003).

Several species mentioned above, including *Paratetillabacca*, *Spirastrelladecumbens*, and Petrosia (Petrosia) hoeksemai, have demonstrated preferences for sedimented environments (Putchakarn 2011; Schönberg 2021). Although psammobiotic species typically exhibit an affinity for sedimented habitats (Schönberg 2016), sediment presence can exert negative pressures on sponge communities. Specifically, when subjected to elevated concentrations of suspended sediment, sponge taxa can exhibit diminished pumping activity and reduced feeding efficiency (Lohrer et al. 2006). Moreover, there may be alterations in their respiration rates (Pineda et al. 2017) and tissue abrasion (Nava and Carballo 2013). Such physiological stressors can culminate in partial mortality and compromised survival rates. A decline in sponge abundance, biomass, and species diversity has the potential to instigate cascading effects on broader marine ecosystems (Bell 2008).

## ﻿Conclusions

In the littoral area, sponges predominantly colonize coral matrices and other hard substrates. Our recent investigation uncovers previously undocumented occurrences, including potentially new taxa, within the sponge community residing in the Sulawesi Sea/Makassar Strait marine ecoregion, particularly at the Spermonde Archipelago, SW Sulawesi. Noteworthy findings include the identification of 15 new records for the marine ecoregion, bringing the total to 143 species on the checklist, not including four potentially novel species. The sponge assemblage within this archipelago presents a rich and intricate biodiversity, underscoring an immediate imperative for comprehensive characterization. Rigorous examination coupled with molecular analysis of specimens is essential to ensure description of the entire species set.

## Supplementary Material

XML Treatment for Clathrina rodriguesensis

XML Treatment for Janusya tubuloreticulosa

XML Treatment for Leucaltis nodusgordii

XML Treatment for Spirastrella aff. decumbens

XML Treatment for Callyspongia (Cladochalina) johannesthielei

XML Treatment for Haliclona (Gellius) cymaeformis

XML Treatment for Haliclona (Reniera) venusta

XML Treatment for Haliclona (Soestella) elegantia

XML Treatment for Haliclona (Soestella)

XML Treatment for Haliclona (Soestella)

XML Treatment for Amphimedon paraviridis

XML Treatment for Niphates nitida

XML Treatment for Petrosia (Petrosia) hoeksemai

XML Treatment for Lissodendoryx (Waldoschmittia) schmidti

XML Treatment for Iotrochota baculifera

XML Treatment for Clathria (Thalysias) reinwardti

XML Treatment for Stylissa massa

XML Treatment for Halichondria (Halichondria) cartilaginea

XML Treatment for Topsentia indica

XML Treatment for Suberites

XML Treatment for Terpios hoshinota

XML Treatment for Ecionemia acervus

XML Treatment for Geodia

XML Treatment for Paratetilla bacca

XML Treatment for Lamellodysidea herbacea

XML Treatment for Ircinia schulzei

XML Treatment for Phyllospongia foliascens

## ﻿Additional information

### Author ORCIDs

Singgih Afifa Putra https://orcid.org/0000-0003-1945-7513

Rohani Ambo-Rappe https://orcid.org/0000-0001-9276-7492

### Data availability

All of the data that support the findings of this study are available in the main text or Supplementary Information.

## Supplementary materials

Supplementary material 1 Sampling sites of sponge (Porifera) collections from shallow-subtidal habitat of the Spermonde Archipelago, Indonesia Data type docx https://binary.pensoft.net/file/1106249This dataset is made available under the Open Database License (http://opendatacommons.org/licenses/odbl/1.0/). The Open Database License (ODbL) is a license agreement intended to allow users to freely share, modify, and use this Dataset while maintaining this same freedom for others, provided that the original source and author(s) are credited.Singgih Afifa Putra, Rohani Ambo-Rappe, Jamaluddin Jompa, Nicole J. de Voogd

Supplementary material 2 checklist of Porifera from Sulawesi Sea/Makassar Strait marine ecoregion with updates based on the current study Data type docx https://binary.pensoft.net/file/1106250This dataset is made available under the Open Database License (http://opendatacommons.org/licenses/odbl/1.0/). The Open Database License (ODbL) is a license agreement intended to allow users to freely share, modify, and use this Dataset while maintaining this same freedom for others, provided that the original source and author(s) are credited.Singgih Afifa Putra, Rohani Ambo-Rappe, Jamaluddin Jompa, Nicole J. de Voogd

Supplementary material 3 List of sponge (Porifera) species examined in this study with locations and environmental condition in the Spermonde Archipelago, Indonesia Data type docx https://binary.pensoft.net/file/1106251This dataset is made available under the Open Database License (http://opendatacommons.org/licenses/odbl/1.0/). The Open Database License (ODbL) is a license agreement intended to allow users to freely share, modify, and use this Dataset while maintaining this same freedom for others, provided that the original source and author(s) are credited.Singgih Afifa Putra, Rohani Ambo-Rappe, Jamaluddin Jompa, Nicole J. de Voogd
