# Supplementary material for: ﻿Preliminary study of marine sponges (Porifera) in the littoral of Spermonde Archipelago, Indonesia
Source: Zookeys. 2024 Aug 1;1208:275–313. doi: 10.3897/zookeys.1208.113603 (PMC11310579; doi:10.3897/zookeys.1208.113603)
Supplement: Supplementary material 1 — Sampling sites of sponge (Porifera) collections from shallow-subtidal habitat of the Spermonde Archipelago, Indonesia [file zookeys-1208-275_article-113603__-s001.docx]

Supplementary Table 1. Sampling sites of sponge (Porifera) collections from shallow-subtidal habitat of the Spermonde Archipelago, Indonesia. * NJdeV collections.

| **Locality** | **Coordinates** | **Survey time** |
| --- | --- | --- |
| Kayangan Island (West) | 05°06'51.40" S, 119°23'50.80" E | 2020 |
| Gusung Tallang | 05°07'20.33" S, 119°23'37.19" E | 2021 |
| Samalona (Northwest) | 05º07’21.00” S, 119º20’29.70” E | 2020 |
| Samalona (Southwest) | 05º07’36.69” S, 119º20’24.46” E | 2020 |
| Samalona* | 05º07’21.17” S, 119º20’21.22” E | 2018 |
| Kudingareng Keke* | 05°06'28.00" S ,119°17'09.90" E | 2018 |
| Barangbaringan* | 05°03'15.20" S, 119°25'22.90" E | 2018 |
| Lumulumu* | 04°58'25.70" S, 119°12'43.80" E | 2018 |
| Badi* | 04°58'09.10" S, 119°16'58.80" E | 2018 |
| Langkai* | 05°01'44.58" S, 119°05'08.79" E | 2018 |
