# Supplementary material for: ﻿Preliminary study of marine sponges (Porifera) in the littoral of Spermonde Archipelago, Indonesia
Source: Zookeys. 2024 Aug 1;1208:275–313. doi: 10.3897/zookeys.1208.113603 (PMC11310579; doi:10.3897/zookeys.1208.113603)
Supplement: Supplementary material 2 — checklist of Porifera from Sulawesi Sea/Makassar Strait marine ecoregion with updates based on the current study [file zookeys-1208-275_article-113603__-s002.docx]

Supplementary Table 2. A checklist of Porifera from Sulawesi Sea/Makassar Strait marine ecoregion with updates based on the current study.

| **No** | **Taxa** | **Sources** |
| --- | --- | --- |
|  | **CALCAREA** |  |
|  | **Calcaronea** |  |
|  | **Leucosolenida: Amphoriscidae** |  |
| 1 | *Paraleucilla saccharata* (Haeckel, 1872) | (van Soest and de Voogd 2015) |
|  | **Leucosolenida: Heteropiidae** |  |
| 2 | *Heteropia minor* Burton, 1930 | (van Soest and de Voogd 2015) |
| 3 | *Sycettusa sibogae* (Burton, 1930) | (Burton 1930) |
|  | **Leucosolenida: Jenkinidae** |  |
| 4 | *Anamixilla torresi* Poléjaeff, 1883 | (van Soest and de Voogd 2015) |
| 5 | *Uteopsis argentea* (Poléjaeff, 1883) | (van Soest and de Voogd 2015) |
|  | **Calcinea** |  |
|  | **Clathrinida: Clathrinidae** |  |
| 6 | *Clathrina chrysea* Borojevic & Klautau, 2000 | (van Soest and de Voogd 2015) |
| 7 | *Clathrina macleayi* (Lendenfeld, 1885) | (Burton 1930) |
| 8 | *Clathrina purpurea* van Soest & de Voogd, 2015 | (van Soest and de Voogd 2015) |
| 9 | *Clathrina stipitata* (Dendy, 1891) | (van Soest and de Voogd 2015) |
| 10 | *Clathrina rodriguesensis* van Soest & de Voogd, 2018 | This study |
| 11 | *Janusya tubuloreticulosa* (van Soest & de Voogd, 2015) | This study |
|  | **Clathrinida: Dendyidae** |  |
| 12 | *Ascandra chrysops* (van Soest & de Voogd, 2015) | (van Soest and de Voogd 2015) |
| 13 | *Neoernsta indonesiae* (van Soest & de Voogd, 2015) | (van Soest and de Voogd 2015) |
|  | **Clathrinida: Leucaltidae** |  |
| 14 | *Leucaltis nodusgordii* (Poléjaeff, 1883) | (van Soest and de Voogd 2015) |
|  | **Clathrinida: Leucascidae** |  |
| 15 | *Leucascus flavus* Cavalcanti, Rapp & Klautau, 2013 | (Cavalcanti et al. 2013) |
|  | **Clathrinida: Leucettidae** |  |
| 16 | *Leucetta chagosensis* Dendy, 1913 | (Cavalcanti et al. 2013); (van Soest and de Voogd 2015) |
| 17 | *Leucetta microraphis* Haeckel, 1872 | (van Soest and de Voogd 2015) |
| 18 | *Pericharax orientalis* van Soest & de Voogd, 2015 | (van Soest and de Voogd 2015) |
|  | **Clathrinida: Levinellidae** |  |
| 19 | *Burtonulla sibogae* Borojevic & Boury-Esnault, 1986 | (van Soest and de Voogd 2015) |
|  | **DEMOSPONGIAE** |  |
|  | **Heteroscleromorpha** |  |
|  | **Axinellida: Axinellidae** |  |
| 20 | *Axinella aruensis* (Hentschel, 1912) | (Alvarez et al. 2016) |
| 21 | *Phycopsis pesgalli* Alvarez, de Voogd & van Soest, 2016 | (Alvarez et al. 2016) |
| 22 | *Ptilocaulis spiculifer* (Lamarck, 1814) | (Alvarez et al. 2016) |
|  | **Axinellida: Raspailiidae (Echinodictyinae)** |  |
| 23 | *Echinodictyum cavernosum* Thiele, 1899 | (Thiele 1899) |
|  | **Biemnida: Rhabderemiidae** |  |
| 24 | *Rhabderemia acanthostyla* Thomas, 1968 | (van Soest and Hooper 1993) |
|  | **Bubarida: Bubaridae** |  |
| 25 | *Phakellia atypica* Lévi, 1961 | (Alvarez et al. 2016) |
|  | **Bubarida: Dictyonellidae** |  |
| 26 | *Acanthella cavernosa* Dendy, 1922 | (Dendy 1922) |
|  | **Clionaida: Clionaidae** |  |
| 27 | *Cliona albimarginata* Calcinai, Bavestrello & Cerrano, 2005 | (Calcinai et al. 2005) |
| 28 | *Cliona favus* Calcinai, Bavestrello & Cerrano, 2005 | (Calcinai et al. 2005) |
| 29 | *Cliona liangae* Calcinai, Bavestrello & Cerrano, 2005 | (Calcinai et al. 2005) |
| 30 | *Cliona mucronata* Sollas, 1878 | (Calcinai et al. 2005) |
| 31 | *Cliona utricularis* Calcinai, Bavestrello & Cerrano, 2005 | (Calcinai et al. 2005) |
| 32 | *Cliothosa dichotoma* (Calcinai, Cerrano, Sarà & Bavestrello, 2000) | (Calcinai et al. 2005) |
| 33 | *Spheciospongia inconstans* (Dendy, 1887) | (Thiele 1899) |
|  | **Clionaida: Placospongiidae** |  |
| 34 | *Placospongia melobesioides* Gray, 1867 | (Thiele 1899); (Vosmaer and Vernhout 1902) |
|  | **Clionaida: Spirastrellidae** |  |
| 35 | *Spirastrella cunctatrix* Schmidt, 1868 | (Vosmaer 1911) |
| 36 | *Spirastrella* aff. *decumbens* Ridley, 1884 | This study |
|  | **Haplosclerida: Callyspongiidae** |  |
| 37 | *Callyspongia* (*Cladochalina*) *johannesthielei* van Soest & Hooper, 2020 | (Thiele 1899) |
| 38 | *Callyspongia* (*Euplacella*) *biru* de Voogd, 2004 | (de Voogd 2004) |
|  | **Haplosclerida: Chalinidae** |  |
| 39 | *Chalinula milnei* (de Laubenfels, 1954) | (Hoeksema et al. 2014) |
| 40 | *Chalinula nematifera* (de Laubenfels, 1954) | (Rossi et al. 2015) |
| 41 | *Cladocroce aculeata* Pulitzer-Finali, 1982 | (Bertolino et al. 2023) |
| 42 | *Cladocroce burapha* Putchakarn, de Weerdt, Sonchaeng & van Soest, 2004 | (Bertolino et al. 2023) |
| 43 | *Haliclona* (*Flagellia*) *hamata* (Thiele, 1903) | (Calcinai et al. 2013) |
| 44 | *Haliclona* (*Flagellia*) *indonesiae* van Soest, 2017 | (van Soest 2017) |
| 45 | *Haliclona* (*Gellius*) *cymaeformis* (Esper, 1806) | This study |
| 46 | *Haliclona* (*Halichoclona*) *vanderlandi* de Weerdt & van Soest, 2001 | (de Weerdt and van Soest 2001) |
| 47 | *Haliclona* (*Reniera*) *venusta* (Bowerbank, 1875) | This study |
| 48 | *Haliclona* (*Soestella*) *elegantia* (Bowerbank, 1875) | This study |
|  | **Haplosclerida: Niphatidae** |  |
| 49 | *Amphimedon anastomosa* Calcinai, Bastari, Bertolino & Pansini, 2017 | (Calcinai et al. 2017b) |
| 50 | *Amphimedon denhartogi* de Voogd, 2003 | (de Voogd 2003) |
| 51 | *Amphimedon paraviridis* Fromont, 1993 | This study |
| 52 | *Gelliodes spinosella* Thiele, 1899 | (Thiele 1899) |
| 53 | *Niphates laminaris* Calcinai, Bastari, Bertolino & Pansini, 2017 | (Calcinai et al. 2017b) |
| 54 | *Niphates nitida* Fromont, 1993 | This study |
|  | **Haplosclerida: Petrosiidae** |  |
| 55 | *Acanthostrongylophora ingens* (Thiele, 1899) | (Thiele 1899); (Esposito et al. 2019) |
| 56 | *Neopetrosia chaliniformis* (Thiele, 1899) | (Thiele 1899) |
| 57 | *Neopetrosia contignata* (Thiele, 1899) | (Thiele 1899) |
| 58 | *Neopetrosia rava* (Thiele, 1899) | (Thiele 1899) |
| 59 | *Petrosia* (*Petrosia*) *alfiani* de Voogd & van Soest, 2002 | (de Voogd and van Soest 2002) |
| 60 | Petrosia (*Petrosia*) *hoeksemai* de Voogd & van Soest, 2002 | (de Voogd and van Soest 2002) |
| 61 | *Petrosia* (*Petrosia*) *lignosa* Wilson, 1925 | (de Voogd and van Soest 2002) |
| 62 | *Petrosia* (*Petrosia*) *nigricans* Lindgren, 1897 | (de Voogd and van Soest 2002); (Thiele 1899) |
| 63 | *Petrosia* (*Petrosia*) *plana* Wilson, 1925 | (de Voogd and van Soest 2002) |
| 64 | *Petrosia* (*Strongylophora*) *corticata* (Wilson, 1925) | (de Voogd and van Soest 2002) |
| 65 | *Petrosia* (*Strongylophora*) *strongylata* Thiele, 1903 | (de Voogd and van Soest 2002) |
|  | **Haplosclerida: Phloeodictyidae** |  |
| 66 | *Oceanapia media* (Thiele, 1899) | (Thiele 1899) |
| 67 | *Siphonodictyon maldiviense* (Calcinai, Cerrano, Sarà & Bavestrello, 2000) | (Calcinai et al. 2007) |
| 68 | *Siphonodictyon microterebrans* (Calcinai, Cerrano & Bavestrello, 2007) | (Calcinai et al. 2007) |
|  | **Poecilosclerida: Acarnidae** |  |
| 69 | *Acarnus bicladotylotus* Hoshino, 1981 | (van Soest et al. 1991) |
|  | **Poecilosclerida: Chondropsidae** |  |
| 70 | *Chondropsis subtilis* Calcinai, Bavestrello, Bertolino, Pica, Wagner & Cerrano, 2013 | (Calcinai et al. 2013) |
|  | **Poecilosclerida: Coelocarteriidae** |  |
| 71 | *Coelocarteria agglomerans* Azzini, Calcinai & Pansini, 2007 | (Azzini et al. 2007) |
|  | **Poecilosclerida: Coelosphaeridae** |  |
| 72 | *Coelosphaera* (*Coelosphaera*) *navicelligera* (Ridley, 1885) | (Thiele 1899) |
| 73 | *Lissodendoryx* (*Lissodendoryx*) *similis* Thiele, 1899 | (Thiele 1899) |
| 74 | *Lissodendoryx* (*Lissodendoryx*) *ternatensis* (Thiele, 1903) | (Hofman and van Soest 1995) |
| 75 | *Lissodendoryx* (*Waldoschmittia*) *schmidti* (Ridley, 1884) | This study |
|  | **Poecilosclerida: Desmacididae** |  |
| 76 | *Desmapsamma vervoorti* van Soest, 1998 | (van Soest 1998) |
|  | **Poecilosclerida: Esperiopsidae** |  |
| 77 | *Esperiopsis challengeri* (Ridley, 1885) | (Ridley 1885) |
|  | **Poecilosclerida: Hymedesmiidae** |  |
| 78 | *Hymedesmia* (*Hymedesmia*) *spinata* Calcinai, Bavestrello, Bertolino, Pica, Wagner & Cerrano, 2013 | (Calcinai et al. 2013) |
| 79 | *Hymedesmia* (*Stylopus*) *perlucida* Calcinai, Bavestrello, Bertolino, Pica, Wagner & Cerrano, 2013 | (Calcinai et al. 2013) |
|  | **Poecilosclerida: Iotrochotidae** |  |
| 80 | *Iotrochota baculifera* Ridley, 1884 | (Thiele 1899) |
|  | **Poecilosclerida: Microcionidae (Microcioninae)** |  |
| 81 | *Clathria* (*Thalysias*) *erecta* (Thiele, 1899) | (Thiele 1899) |
| 82 | *Clathria* (*Thalysias*) *reinwardti* Vosmaer, 1880 | (Thiele 1899) |
| 83 | *Clathria* (*Thalysias*) *topsenti* (Thiele, 1899) | (Thiele 1899) |
| 84 | *Clathria* (*Thalysias*) *vulpina* (Lamarck, 1814) | (Hooper 1996) |
|  | **Poecilosclerida: Mycalidae** |  |
| 85 | *Mycale* (*Aegogropila*) *furcata* Calcinai, Bavestrello, Bertolino, Pica, Wagner & Cerrano, 2013 | (Calcinai et al. 2013) |
| 86 | *Mycale* (*Aegogropila*) *orientalis* (Topsent, 1897) | (van Soest et al. 2021) |
| 87 | *Mycale* (*Arenochalina*) *regularis* Wilson, 1925 | (van Soest et al. 2021) |
| 88 | *Mycale* (Carmia) *fungiaphila* van Soest, Aryasari & de Voogd, 2021 | (van Soest et al. 2021) |
| 89 | *Mycale* (*Carmia*) *lissochela* Bergquist, 1965 | (van Soest et al. 2021) |
| 90 | *Mycale* (*Carmia*) *phyllophila* Hentschel, 1911 | (van Soest et al. 2021) |
| 91 | *Mycale* (*Carmia*) *tubiporicola* Van Soest, Aryasari & de Voogd, 2021 | (van Soest et al. 2021) |
| 92 | *Mycale* (*Kerasemna*) *humilis* (Thiele, 1903) | (Calcinai et al. 2006; van Soest et al. 2021) |
| 93 | *Mycale* (*Mycale*) *crassissima* (Dendy, 1905) | (van Soest et al. 2021) |
| 94 | *Mycale* (*Mycale*) *dendyi* (Row, 1911) | (van Soest et al. 2021) |
| 95 | *Mycale* (*Mycale*) *grandis* Gray, 1867 | (van Soest et al. 2021) |
| 96 | Mycale (*Naviculina*) *cleistochela* Vacelet & Vasseur, 1971 | (van Soest et al. 2021) |
| 97 | Mycale (*Naviculina*) *obscura* (Carter, 1882) | (van Soest et al. 2021) |
| 98 | Mycale (*Paresperella*) *sceptroides* van Soest, Aryasari & de Voogd, 2021 | (van Soest et al. 2021) |
| 99 | *Mycale* (*Zygomycale*) *parishii* (Bowerbank, 1875) | (van Soest et al. 2021) |
|  | **Poecilosclerida: Podospongiidae** |  |
| 100 | *Podospongia colini* Sim-Smith & Kelly, 2011 | (Sim-Smith and Kelly 2011) |
|  | **Scopalinida: Scopalinidae** |  |
| 101 | *Stylissa carteri* (Dendy, 1889) | (Eder et al. 1999) |
| 102 | *Stylissa massa* (Carter, 1887) | (Thiele 1899) |
| 103 | *Svenzea devoogdae* Alvarez, van Soest & Rützler, 2002 | (Alvarez et al. 2002) |
|  | **Suberitida: Halichondriidae** |  |
| 104 | *Axinyssa valida* (Thiele, 1899) | (Thiele 1899) |
| 105 | *Halichondria* (*Halichondria*) *cartilaginea* (Esper, 1797) | This study |
| 106 | *Topsentia indica* Hentschel, 1912 | This study |
|  | **Suberitida: Suberitidae** |  |
| 107 | *Aaptos lobata* Calcinai, Bastari, Bertolino & Pansini, 2017 | (Calcinai et al. 2017b) |
| 108 | *Terpios hoshinota* Rützler & Muzik, 1993 | (van der Ent et al. 2016) |
|  | **Tethyida: Hemiasterellidae** |  |
| 109 | *Liosina paradoxa* Thiele, 1899 | (Thiele 1899) |
|  | **Tethyida: Tethyidae** |  |
| 110 | *Tethytimea tylota* (Hentschel, 1912) | (Calcinai et al. 2017b) |
|  | **Tetractinellida (Astrophorina): Ancorinidae** |  |
| 111 | *Dercitus* (*Stoeba*) *bangkae* Calcinai, Bastari, Makapedua & Cerrano, 2017 | (Calcinai et al. 2017a) |
| 112 | *Ecionemia acervus* Bowerbank, 1862 | (Thiele 1899) |
| 113 | *Rhabdastrella distincta* (Thiele, 1900) | (Calcinai et al. 2017b) |
|  | **Tetractinellida (Astrophorina): Geodiidae (Erylinae)** |  |
| 114 | *Melophlus sarasinorum* Thiele, 1899 | (Thiele 1899) |
|  | **Tetractinellida (Astrophorina): Theonellidae** |  |
| 115 | *Theonella swinhoei* Gray, 1868 | (Thiele 1899) |
|  | **Tetractinellida (Spirophorina): Tetillidae** |  |
| 116 | *Acanthotetilla celebensis* de Voogd & van Soest, 2007 | (de Voogd and van Soest 2007) |
| 117 | *Cinachyrella australiensis* (Carter, 1886) | (Thiele 1899) |
| 118 | *Paratetilla bacca* (Selenka, 1867) | This study |
| 119 | *Tetilla disigmata* Lévi, 1964 | (Lévi 1964) |
|  | **Keratosa** |  |
|  | **Dictyoceratida: Dysideidae** |  |
| 120 | *Lamellodysidea herbacea* (Keller, 1889) | This study |
|  | **Dictyoceratida: Irciniidae** |  |
| 121 | *Ircinia colossa* Calcinai, Bastari, Bertolino & Pansini, 2017 | (Calcinai et al. 2017b) |
| 122 | *Ircinia schulzei* (Dendy, 1905) | This study |
| 123 | *Psammocinia alba* Calcinai, Bastari, Bertolino & Pansini, 2017 | (Calcinai et al. 2017b) |
|  | **Dictyoceratida: Thorectidae (Phyllospongiinae)** |  |
| 124 | *Phyllospongia foliascens* (Pallas, 1766) | This study |
| 125 | *Phyllospongia palmata* Thiele, 1899 | (Thiele 1899) |
| 126 | *Phyllospongia papyracea* (Esper, 1806) | (Thiele 1899) |
|  | **Dictyoceratida: Thorectidae (Thorectinae)** |  |
| 127 | *Dactylospongia elegans* (Thiele, 1899) | (Thiele 1899) |
| 128 | *Hyrtios reticulatus* (Thiele, 1899) | (Thiele 1899) |
|  | **Verongimorpha** |  |
|  | **Verongiida: Pseudoceratinidae** |  |
| 129 | *Pseudoceratina purpurea* (Carter, 1880) | (Brøndsted 1934; Thiele 1899) |
|  | **HEXACTINELLIDA** |  |
|  | **Amphidiscophora** |  |
|  | **Amphidiscosida: Hyalonematidae** |  |
| 130 | *Hyalonema* (*Oonema*) *trifidum* Lévi, 1964 | (Lévi 1964) |
|  | **Amphidiscosida: Pheronematidae** |  |
| 131 | *Ijimalophus reflexus* (Ijima, 1894) | (Ijima 1927) |
| 132 | *Pheronema barbulosclera* Lévi, 1964 | (Lévi 1964) |
| 133 | *Pheronema pilosum* Lévi, 1964 | (Tabachnick and Lévi 2000) |
| 134 | *Semperella similis* Ijima, 1927 | (Ijima 1927) |
|  | **Hexasterophora** |  |
|  | **Lyssacinosida: Euplectellidae (Bolosominae)** |  |
| 135 | *Bolosoma cavum* Ijima, 1927 | (Lévi 1990) |
|  | **Lyssacinosida: Euplectellidae (Corbitellinae)** |  |
| 136 | *Regadrella cylindrica* Ijima, 1927 | (Ijima 1927) |
|  | **Lyssacinosida: Rossellidae (Acanthascinae)** |  |
| 137 | *Staurocalyptus celebesianus* Ijima, 1927 | (Ijima 1927) |
|  | **Lyssacinosida: Rossellidae (Rossellinae)** |  |
| 138 | *Crateromorpha* (*Aulochone*) *pedunculata* (Ijima, 1927) | (Ijima 1927) |
|  | **Sceptrulophora: Euretidae (Euretinae)** |  |
| 139 | *Pararete baliense* Ijima, 1927 | (Ijima 1927) |
| 140 | *Pararete kangeanganum* Ijima, 1927 | (Ijima 1927) |
|  | **Sceptrulophora: Farreidae** |  |
| 141 | *Aspidoscopulia furcillata* (Lévi, 1990) | (Lévi 1990) |
| 142 | *Farrea occa ouwensi* Ijima, 1927 | (Ijima 1927) |
|  | **HOMOSCLEROMORPHA** |  |
|  | **Homosclerophorida: Plakinidae** |  |
| 143 | *Plakortis bergquistae* Muricy, 2011 | (Muricy 2011) |

**References**

Alvarez B, van Soest RWM, Rützler K (2002) *Svenzea*, a new genus of Dictyonellidae (Porifera: Demospongiae) from tropical reef environments, with description of two new species. Contributions to Zoology 71: 171–176. https://doi.org/10.1163/18759866-07104007

Alvarez B, de Voogd NJ, van Soest RWM (2016) Sponges of the family Axinellidae (Porifera: Demospongiae) in Indonesia. Zootaxa 4137: 451–477. https://doi.org/10.11646/zootaxa.4137.4.1

Azzini F, Calcinai B, Pansini M (2007) A new species of Coelocarteria (Porifera: Demospongiae) from Sulawesi, Indonesia. Journal of the Marine Biological Association of the United Kingdom 87: 1349–1353. https://doi.org/10.1017/S0025315407058365

Bertolino M, Cerrano C, Bavestrello G, Thung DC, Núñez-Pons L, Rispo F, Efremova J, Mazzella V, Makapedua DM, Calcinai B (2023) New Insight into the Genus *Cladocroce* (Porifera, Demospongiae) Based on Morphological and Molecular Data, with the Description of Two New Species. Journal of Marine Science and Engineering 11: 1240. https://doi.org/10.3390/jmse11061240

Brøndsted HV (1934) Sponges. In: Van Straelen V (Ed.), Résultats Scientifiques du Voyage aux Indes Orientales Néerlandaises de LL. AA. RR. le Prince et la Princesse Léopold de Belgique. Mémoires du Musée royal d’histoire naturelle de Belgique, Hors série, 1–27.

Burton M (1930) The Porifera of the Siboga Expedition. III. Calcarea. Siboga-Expeditie. Uitkomsten op zoologisch, botanisch, oceanographisch en geologisch gebied verzameld in Nederlandsch Oost-lndië 1899-1900aan boord H.M. ‘Siboga’’ onder commando van Luitenant ter Zee 1e kl. G.F. Tydeman. 9 (Monographie VIa2)’: 1–18.

Calcinai B, Bavestrello G, Cerrano C (2005) Excavating sponge species from the Indo-Pacific Ocean. Zoological Studies 44: 5–18.

Calcinai B, Cerrano C, Bavestrello G (2007) Three new species and one re-description of *Aka*. Journal of the Marine Biological Association of the United Kingdom 87: 1355–1365. https://doi.org/10.1017/S0025315407058377

Calcinai B, Bastari A, Makapedua DM, Cerrano C (2017a) Mangrove sponges from Bangka Island (North Sulawesi, Indonesia) with the description of a new species. Journal of the Marine Biological Association of the United Kingdom 97: 1417–1422. https://doi.org/10.1017/S0025315416000710

Calcinai B, Cerrano C, Totti C, Romagnoli T, Bavestrello G (2006) Symbiosis of *Mycale (Mycale) vansoesti* sp. nov. (Porifera, Demospongiae) with a coralline alga from North Sulawesi (Indonesia). Invertebrate Biology 125: 195–204. https://doi.org/10.1111/j.1744-7410.2006.00052.x

Calcinai B, Bavestrello G, Bertolino M, Pica D, Wagner D, Cerrano C (2013) Sponges associated with octocorals in the Indo-Pacific, with the description of four new species. Zootaxa 3617: 1–61. https://doi.org/10.11646/zootaxa.3617.1.1

Calcinai B, Bastari A, Bavestrello G, Bertolino M, Horcajadas SB, Pansini M, Makapedua DM, Cerrano C (2017b) Demosponge diversity from North Sulawesi, with the description of six new species. ZooKeys 680: 105–150. https://doi.org/10.3897/zookeys.680.12135

Cavalcanti FF, Rapp HT, Klautau M (2013) Taxonomic revision of *Leucascus* Dendy, 1892 (Porifera: Calcarea) with revalidation of *Ascoleucetta* Dendy & Frederick, 1924 and description of three new species. Zootaxa 3619: 275–314. https://doi.org/10.11646/zootaxa.3619.3.3

Dendy A (1922) Report on the Sigmatotetraxonida collected by H.M.S.‘Sealark’’ in the Indian Ocean. In: Reports of the Percy Sladen Trust Expedition to the Indian Ocean in 1905, Vol. 7.’ Transactions of the Linnean Society of London 18: 1–164, pls1-18. Available from: <http://www.marinespecies.org/aphia.php?p=sourcedetails&id=7412> [accessed 15-09-2021]

Eder C, Proksch P, Wray V, Steube K, Bringmann G, van Soest RWM, Sudarsono, Ferdinandus E, Pattisina LA, Wiryowidagdo S, Moka W (1999) New Alkaloids from the Indopacific Sponge *Stylissa carteri*. Journal of Natural Products 62: 184–187. https://doi.org/10.1021/np980315g

van der Ent E, Hoeksema BW, de Voogd NJ (2016) Abundance and genetic variation of the coral-killing cyanobacteriosponge *Terpios hoshinota* in the Spermonde Archipelago, SW Sulawesi, Indonesia. Journal of the Marine Biological Association of the United Kingdom 96: 453–463. https://doi.org/10.1017/S002531541500034X

Esposito G, Mai LH, Longeon A, Mangoni A, Durieu E, Meijer L, Van Soest R, Costantino V, Bourguet-Kondracki M-L (2019) A Collection of Bioactive Nitrogen-Containing Molecules from the Marine Sponge *Acanthostrongylophora ingens*. Marine Drugs 17: 472. https://doi.org/10.3390/md17080472

Hoeksema BW, Dekker F, de Voogd NJ (2014) Free-living mushroom corals strike back by overtopping a coral-killing sponge. Marine Biodiversity 44: 3–4. https://doi.org/10.1007/s12526-013-0188-x

Hofman CC, van Soest RWM (1995) *Lissodendoryx* species of the Indo-Malayan Archipelago (Demospongiae: Poecilosclerida). Beaufortia 45: 77–103.

Hooper JNA (1996) Revision of Microcionidae (Porifera: Poecilosclerida: Demospongiae), with Description of Australian Species. Memoirs of the Queensland Museum 40: 1–626.

Ijima I (1927) The Hexactinellida of the Siboga Expedition. In: Weber M (Ed.), Uitkomsten op zoologisch, botanisch, oceanographisch et geologisch Gebied versameld in Nederlandsk Oost-Indie 1899–1900. E. J. Brill, Leiden, 1–383.

Lévi C (1964) Spongiaires des zones bathyale, abyssale et hadale. Galathea Report. Scientific Results of The Danish Deep-Sea Expedition Round the World, 1950-52 7: 63–112, pls II–XI. Available from: <http://marinespecies.org/aphia.php/aphia.php?p=sourcedetails&id=7855> [accessed 14-09-2021]

Lévi C (1990) *Claviscopulia furcillata* n. sp. et autres Hexactinellida (Porifera) des mers indonésiennes. Bulletin du Muséum d’Histoire naturelle 12: 277–290. Available from: <http://www.marinespecies.org/porifera/porifera.php?p=sourcedetails&id=7865> [accessed 11-12-2021]

Muricy G (2011) Diversity of Indo-Australian *Plakortis* (Demospongiae: Plakinidae), with description of four new species. Journal of the Marine Biological Association of the United Kingdom 91: 303–319. https://doi.org/10.1017/S0025315410000743

Ridley SO (1885) Monaxonida. In: Tizard TH, Moseley HN, Buchanan JY, Murray J (Eds), Narrative of the cruise of H.M.S. Challenger, with a general account of the scientific results of the Expedition. Reports of the Scientific Results of the Voyage of the Challenger., 569–573.

Rossi G, Montori S, Cerrano C, Calcinai B (2015) The coral killing sponge *Chalinula nematifera* (Porifera: Haplosclerida) along the eastern coast of Sulawesi Island (Indonesia). Italian Journal of Zoology 82: 143–148. https://doi.org/10.1080/11250003.2014.994046

Sim-Smith C, Kelly M (2011) Two new genera in the family Podospongiidae (Demospongiae: Poecilosclerida) with eight new Western Pacific species. Zootaxa 54: 32–54. https://doi.org/10.11646/zootaxa.2976.1.3

van Soest RWM (1998) A new sponge *Desmapsamma vervoorti* spec. nov. (Poecilosclerida: Desmacididae) from Indonesia. Zoologische Verhandelingen 323: 427–434. Available from: <http://www.repository.naturalis.nl/document/149038> [accessed 19-10-2019]

van Soest RWM (2017) *Flagellia*, a new subgenus of *Haliclona* (Porifera, Haplosclerida). European Journal of Taxonomy 2017: 1–48. https://doi.org/10.5852/ejt.2017.351

van Soest RWM, Hooper JNA (1993) Taxonomy, phylogeny and biogeography of the marine sponge genus *Rhabderemia* Topsent, 1890 (Demospongiae, Poecilosclerida). In: Uriz MJ, Rützler K (Eds), Recent Advance in Ecology and Systematics of Sponges. Scientia Marina. Instituto de Ciencias del Mar - CSIC, Barcelona, 319–351.

van Soest RWM, de Voogd NJ (2015) Calcareous sponges of Indonesia. Zootaxa 3951: 1–105. https://doi.org/10.11646/zootaxa.3951.1.1

van Soest RWM, Hooper JNA, Hiemstra F (1991) Taxonomy, phylogeny and biogeography of the marine sponge genus *Acarnus* (Porifera: Poecilosclerida). Beaufortia 42: 49–88. Available from: <https://repository.naturalis.nl/pub/504942> [accessed 08-09-2021]

van Soest RWM, Aryasari R, de Voogd NJ (2021) *Mycale* species of the tropical Indo-West Pacific (Porifera, Demospongiae, Poecilosclerida). Zootaxa 4912: 1–212. https://doi.org/10.11646/zootaxa.4912.1.1

Tabachnick KR, Lévi C (2000) Porifera Hexactinellida: Amphidiscophora off New Caledonia. In: Crosnier A (Ed.), Résultats des Campagnes MUSORSTOM 21. Mémoires du Muséum national d’Histoire naturelle. Série A, Zoologie. Publications Scientifiques du Muséum, Paris, 53–140. Available from: <https://www.vliz.be/en/maps-library?module=ref&refid=18679> [accessed 27-12-2022]

Thiele J (1899) Studien über pazifische Spongien. II. Ueber einige Spongien von Celebes. Original-Abhandlungen aus dem Gesamtgebiete der Zoologie. Stuttgart 24: 1–33. Available from: <http://www.marinespecies.org/porifera/porifera.php?p=sourcedetails&id=8296> [accessed 25-12-2019]

de Voogd NJ (2003) *Amphimedon denhartogi* spec. nov. (Porifera: Haplosclerida) from deep reef habitats in Indonesia. Zoologische Verhandelingen 345: 413–418. Available from: <https://repository.naturalis.nl/pub/220574> [accessed 13-10-2019]

de Voogd NJ (2004) *Callyspongia* (*Euplacella*) *biru* spec. nov. (Porifera: Demospongiae: Haplosclerida) from Indonesia. Zoologische Mededelingen 78: 477–483. Available from: <https://repository.naturalis.nl/pub/214454> [accessed 13-10-2019]

de Voogd NJ, van Soest RWM (2002) Indonesian sponges of the genus *Petrosia* Vosmaer (Demospongiae : Haplosclerida). Zoologische Mededelingen (Leiden) 76: 193–209. Available from: <https://hdl.handle.net/11245/1.197534> [accessed 13-10-2019]

de Voogd NJ, van Soest RWM (2007) *Acanthotetilla celebensis* sp. nov., a new species from North Sulawesi, Indonesia (Porifera: Demospongiae: Spirophorida: Tetillidae). Zootaxa 1397: 25–28. https://doi.org/10.11646/zootaxa.1397.1.3

Vosmaer GCJ (1911) The Porifera of the Siboga-Expedition: 2. The genus *Spirastrella*. In: Weber M (Ed.), Siboga-Expeditie: uitkomsten op zoölogisch, botanisch, oceanographisch en geologisch gebied verzameld in Nederlandsch Oost-Indië 1899-1900 aan boord H.M. Siboga onder commando van Luitenant ter Zee 1e kl. G.F. Tydeman. E.J. Brill, Leyden, The Netherlands, 69: plates I-XIV. Available from <https://www.marinespecies.org/aphia.php?p=sourcedetails&id=8542> [accessed 19-06-2020]

Vosmaer GCJ, Vernhout JH (1902) The Porifera of the Siboga-Expedition. I. The genus *Placospongia*. In: Weber M (Ed.), Siboga-Expeditie. Uitkomsten op zoologisch, botanisch, oceanographisch en geologisch gebied verzameld in Nederlandsch Oost-lndië 1899-1900aan boord H.M. ‘Siboga’’ onder commando van Luitenant ter Zee 1e kl. G.F. Tydeman. 9 (Monographie VIa).’ E. J. Brill, Leiden, 1–17. Available from <https://www.marinespecies.org/aphia.php?p=sourcedetails&id=8548> [accessed 12-04-2020]

de Weerdt WH, van Soest RWM (2001) *Haliclona* (*Halichoclona*) *vanderlandi* spec. nov. (Porifera: Demospongiae: Haplosclerida) from Indonesia. Zoologische Verhandelingen 334: 189–194. Available from: <https://hdl.handle.net/11245/1.182998> [accessed 13-10-2019]
