# Supplementary material for: ﻿Preliminary study of marine sponges (Porifera) in the littoral of Spermonde Archipelago, Indonesia
Source: Zookeys. 2024 Aug 1;1208:275–313. doi: 10.3897/zookeys.1208.113603 (PMC11310579; doi:10.3897/zookeys.1208.113603)
Supplement: Supplementary material 3 — List of sponge (Porifera) species examined in this study with locations and environmental condition in the Spermonde Archipelago, Indonesia [file zookeys-1208-275_article-113603__-s003.docx]

**Supplementary Table 3.** List of sponge (Porifera) species examined in this study with locations and environmental condition in the Spermonde Archipelago, Indonesia.

| **Sample code** | **Species** | **Location** | **Environment** |
| --- | --- | --- | --- |
| CEL035 | *Clathrina rodriguesensis* van Soest & de Voogd, 2018 | Kudingareng Keke | clear |
| CEL001 | *Janusya tubuloreticulosa* (van Soest & de Voogd, 2015) | Samalona | clear |
| CEL005 | *Leucaltis nodusgordii* (Poléjaeff, 1883) | Samalona | clear |
| CEL007 | *Spirastrella* aff. *decumbens* Ridley, 1884 | Langkai | clear |
| SAM002 | *Callyspongia* (*Cladochalina*) *johannesthielei* van Soest & Hooper, 2020 | Samalona | clear |
| SAM304 | *Haliclona* (*Gellius*) *cymaeformis* (Esper, 1806) | Samalona, Kayangan, Gusung Tallang | turbid |
| SAM103 | *Haliclona* (*Reniera*) *venusta* (Bowerbank, 1875) | Samalona | clear |
| KAY015C | *Haliclona* (*Soestella*) *elegantia* (Bowerbank, 1875) | Kayangan, Gusung Tallang | turbid |
| SAM306 | *Haliclona* (*Soestella*) sp1. nov | Samalona | clear |
| KAY017 | *Haliclona* (*Soestella*) sp2. nov. | Kayangan, Gusung Tallang | turbid |
| SAM3XX | *Amphimedon paraviridis* Fromont, 1993 | Samalona | clear |
| KAY015B | *Niphates nitida* Fromont, 1993 | Kayangan | turbid |
| SAM102 | *Petrosia* (*Petrosia*) *hoeksemai* de Voogd & van Soest 2002 | Samalona | clear |
| CEL079 | *Lissodendoryx* (*Waldoschmittia*) *schmidti* (Ridley, 1884) | Lumulumu | clear |
| SAM302 | *Iotrochota baculifera* Ridley, 1884 | Samalona | clear |
| SAM301 | *Clathria* (*Thalysias*) *reinwardti* Vosmaer, 1880 | Samalona, Gusung Tallang | turbid |
| SAM206 | *Stylissa massa* (Carter, 1887) | Samalona | clear |
| CEL025 | *Halichondria* (*Halichondria*) *cartilaginea* (Esper, 1797) | Badi | clear |
| CEL011 | *Topsentia indica* Hentschel, 1912. | Langkai | clear |
| SAM307 | *Suberites* sp. nov. | Samalona | clear |
| SAM310 | *Terpios hoshinota* Rützler & Muzik, 1993 | Samalona | clear |
| CEL016 | *Ecionemia acervus* Bowerbank, 1862 | Langkai | clear |
| CEL174 | *Geodia* sp. nov. | Barangbaringan | turbid |
| GUS018 | *Paratetilla bacca* (Selenka, 1867) | Gusung Tallang | turbid |
| SAM311 | *Lamellodysidea herbacea* (Keller, 1889) | Samalona | clear |
| SAM001 | *Ircinia schulzei* (Dendy, 1905) | Samalona | clear |
| SAM104 | *Phyllospongia foliascens* (Pallas, 1766) | Samalona, Gusung Tallang | turbid |
